# Sex Differences in Presentation of Stroke: A Systematic Review and Meta-Analysis

**DOI:** 10.1161/STROKEAHA.120.034040

**Published:** 2021-12-14

**Authors:** Mariam Ali, Hendrikus J.A. van Os, Nelleke van der Weerd, Jan W. Schoones, Martijn W. Heymans, Nyika D. Kruyt, Marieke C. Visser, Marieke J.H. Wermer

**Affiliations:** Department of Neurology, Amsterdam UMC, Vrije Universiteit Amsterdam, the Netherlands (M.A., M.C.V.).; Department of Neurology (H.J.A.v.O., N.v.d.W., N.D.K., M.J.H.W.), Leiden University Medical Center, the Netherlands.; Walaeus Library (J.W.S.), Leiden University Medical Center, the Netherlands.; Department of Clinical Epidemiology and Biostatistics, Amsterdam UMC, the Netherlands (M.W.H.).

**Keywords:** diagnostic errors, intracranial hemorrhages, ischemic attack, transient, ischemic stroke, sex characteristics, signs and symptoms

## Abstract

**Background and Purpose:**

Women have worse outcomes than men after stroke. Differences in presentation may lead to misdiagnosis and, in part, explain these disparities. We investigated whether there are sex differences in clinical presentation of acute stroke or transient ischemic attack.

**Methods:**

We conducted a systematic review and meta-analysis according to the Preferred Reporting Items for Systematic Reviews and Meta-Analyses statement. Inclusion criteria were (1) cohort, cross-sectional, case-control, or randomized controlled trial design; (2) admission for (suspicion of) ischemic or hemorrhagic stroke or transient ischemic attack; and (3) comparisons possible between sexes in ≥1 nonfocal or focal acute stroke symptom(s). A random-effects model was used for our analyses. We performed sensitivity and subanalyses to help explain heterogeneity and used the Newcastle-Ottawa Scale to assess bias.

**Results:**

We included 60 studies (n=582 844; 50% women). In women, headache (pooled odds ratio [OR], 1.24 [95% CI, 1.11–1.39]; I^2^=75.2%; 30 studies) occurred more frequently than in men with any type of stroke, as well as changes in consciousness/mental status (OR, 1.38 [95% CI, 1.19–1.61]; I^2^=95.0%; 17 studies) and coma/stupor (OR, 1.39 [95% CI, 1.25–1.55]; I^2^=27.0%; 13 studies). Aspecific or other neurological symptoms (nonrotatory dizziness and non-neurological symptoms) occurred less frequently in women (OR, 0.96 [95% CI, 0.94–0.97]; I^2^=0.1%; 5 studies). Overall, the presence of focal symptoms was not associated with sex (pooled OR, 1.03) although dysarthria (OR, 1.14 [95% CI, 1.04–1.24]; I^2^=48.6%; 11 studies) and vertigo (OR, 1.23 [95% CI, 1.13–1.34]; I^2^=44.0%; 8 studies) occurred more frequently, whereas symptoms of paresis/hemiparesis (OR, 0.73 [95% CI, 0.54–0.97]; I^2^=72.6%; 7 studies) and focal visual disturbances (OR, 0.83 [95% CI, 0.70–0.99]; I^2^=62.8%; 16 studies) occurred less frequently in women compared with men with any type of stroke. Most studies contained possible sources of bias.

**Conclusions:**

There may be substantive differences in nonfocal and focal stroke symptoms between men and women presenting with acute stroke or transient ischemic attack, but sufficiently high-quality studies are lacking. More studies are needed to address this because sex differences in presentation may lead to misdiagnosis and undertreatment.

## Abstract 2

Supplemental Digital Content is available in the text.

Women with stroke have a higher mortality rate and a worse functional outcome compared with men.^1^ It has been hypothesized that this is, at least in part, due to misdiagnosis with consequent delays to or even deferral of acute or secondary preventive treatment.^2,3^

The higher frequency of misdiagnosis in women may in turn be explained by a higher prevalence of nonfocal or atypical stroke symptoms compared with men.^4–7^ These nonfocal symptoms include confusion, impaired consciousness, headache, generalized weakness, and non-neurological symptoms such as chest pain and palpitations. Nonfocal symptoms could mistakenly be interpreted as symptoms with another pathophysiology than stroke (a so-called stroke mimic) such as a conversion disorder or a migraine attack.^8^ Interestingly, a previous cohort study indicated that women who presented with a transient ischemic attack (TIA) or minor stroke more frequently received a diagnosis of stroke mimic compared with men with similar symptomatology.^3^ However, stroke recurrence rates within 90 days were similar for both sexes, raising the possibility of biases or sex-specific differences in TIA/stroke diagnosis.^9,10^ Several pathophysiological mechanisms could explain sex differences in acute stroke symptoms, including differences in cause of stroke or stroke subtype, presence of comorbidities, or sex aspects resulting in different subjective experiences of symptoms.^11^

We systematically reviewed and meta-analyzed the literature on possible disparities between stroke symptoms in women and men to investigate whether there are sex differences in clinical acute stroke symptoms.

## Methods

This systematic review and meta-analysis was conducted according to the Preferred Reporting Items for Systematic Reviews and Meta-Analyses statement.^12^ Data not published within the article are available from the corresponding author on reasonable request. We systematically searched PubMed, Embase, Emcare, Web of Science, and the Cochrane Library for published articles from their inception until May 2020, to identify studies that reported comparisons between men and women in acute stroke symptomatology. The complete search strategy is provided in Supplemental Material.

### Classification of Stroke or TIA Symptoms

The definition of nonfocal symptoms and focal symptoms was based on a previous classification.^13^ We added lightheadedness to the classification nonfocal symptoms and unilateral numbness, discoordination/ataxia, and paresis/hemiparesis to the classification focal symptoms.

Nonfocal symptoms thus included lightheadedness, mental status change/change in level of consciousness (confusion, altered mentality, mental status change, disorientation, and drowsiness defined as Glasgow Coma Scale [GCS] score ≤14; coma/stupor/loss of consciousness/unconsciousness defined as GCS score ≤8), headache including migraine, aspecific neurological or other neurological symptoms (nonrotatory dizziness and non-neurological symptoms), and atypical symptoms including chest pain, palpitations, shortness of breath, nausea, hiccups, and generalized weakness (Table 1).

**Table 1.**
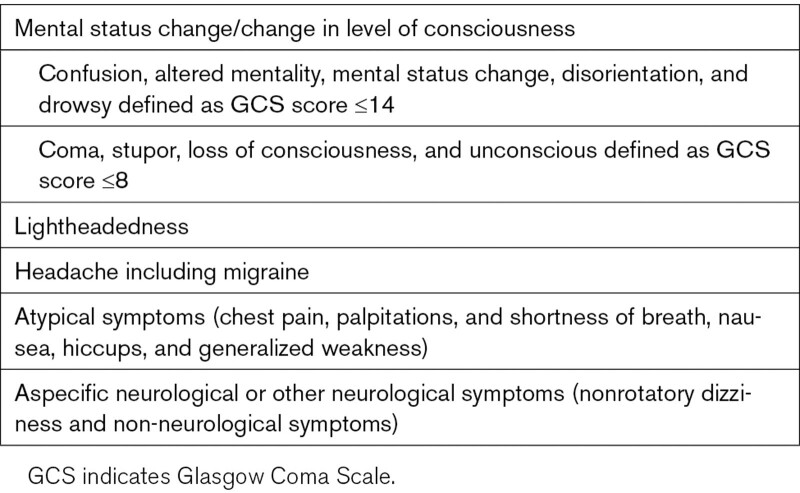
Inclusion of Nonfocal Symptoms

Focal symptoms included unilateral numbness, discoordination/ataxia, paresis/hemiparesis, aphasia, dysarthria, gait disturbance, imbalance, facial weakness, vertigo with or without nausea/vomiting, diplopia, other focal visual disturbances, and pain of neurological origin (face or hemibody pain; Table 2).

**Table 2.**
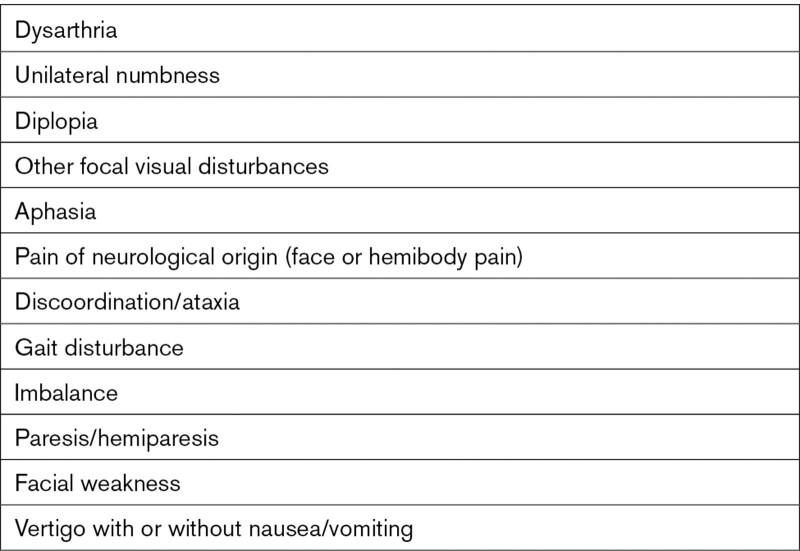
Inclusion of Focal Symptoms

Rare stroke symptoms such as binocular blindness, speaking with a foreign accent, hemiballismus, and alien hand syndrome were excluded.

### Selection Criteria

We applied the following selection criteria for inclusion of studies: (1) patients admitted consecutively because of a diagnosis or suspicion of acute stroke including TIA; (2) use of a cohort, cross-sectional, case-control, or randomized controlled trial design; (3) diagnosis of stroke based on neurological examination and neuroimaging (either computed tomography or magnetic resonance imaging). The diagnosis of TIA had to be based on the anamnesis, neurological examination performed by an emergency medicine doctor or a neurologist (in training) in combination with normal neuroimaging. Patients with TIA had to be examined at specialized TIA centers (TIA clinic or emergency department of a stroke center). A TIA was defined as acute focal neurological symptoms lasting <24 hours. Preferably, analyses for intracerebral hemorrhage (ICH) and subarachnoid hemorrhage (SAH) were performed separately; (4) included patients were ≥18 years of age; (5) presence of ≥1 nonfocal or focal stroke symptoms is quantified in the article; (6) possible differences in stroke symptoms between women and men could be retracted; and (7) articles written in English, Dutch, German, French, Spanish, or Italian. If multiple publications originated from the same cohort, we included the study reporting on the largest number of symptoms by sex. In case similar data were presented, we included the study with the largest population.

### Data Extraction

Two reviewers (M.A. and N.v.d.W.) independently performed 2 rounds of screening: (1) title and abstract and (2) full-text versions of the remaining studies after the first selection. Data extraction forms included details on the study characteristics (publication year, country of study population, setting, study period, stroke subtype, study design, study size, proportion women, and mean or median age). In cases of doubt, a consensus meeting was held with a third reviewer to determine whether articles met the inclusion criteria.

### Assessment of Risk of Bias

The quality assessment of included studies was conducted with the Newcastle-Ottawa Scale.^14,15^ For this study, we developed a customized version of the Newcastle-Ottawa Scale with adjustments for the assessment of stroke and TIA symptoms. Studies were scored low risk, high risk, or possible/unclear on the domains (1) validation of diagnosis, (2) assessment of symptoms, (3) adjustment for confounding, and (4) generalizability (Supplemental Methods).

### Statistical Analysis

Statistical analyses were performed using R. We weighted the log of the odds ratios (ORs) by the inverse of their variance to obtain pooled estimates. We used a random-effects model because we expected heterogeneity to be present due to known differences in stroke patient characteristics and symptom definitions.

We used the sex-specific number of symptom occurrences provided in the study, along with the total number of women or men studied to calculate ORs. We also used this information to calculate the corresponding 95% CIs and the degree of overlap in symptom presentation between men and women in percentages.

In case a patient presented with multiple symptoms, we included this patient in multiple analyses relating to these symptoms. We intended to use the adjusted effect estimates. If not available, the crude effect estimates were pooled.

Meta-analyses per symptom were conducted if at least 2 independent studies quantified the same symptom.

The overall effect estimate and 95% CI of the forest plots per symptom were used to create summary forest plots for both nonfocal and focal symptoms.

Statistical heterogeneity was assessed by the Higgin I^2^ statistics.^16^ We considered study-level estimates to be heterogeneous if the I^2^ value was >50%. I^2^ from 50% to 75% was considered as substantial heterogeneity, and I^2^ >75% was indicated as considerable heterogeneity.

We used funnel plots to examine potential publication bias of symptoms that were reported by at least 10 studies. Furthermore, we intended to perform subgroup analyses for stroke subtypes.

## Results

**Figure 1.**
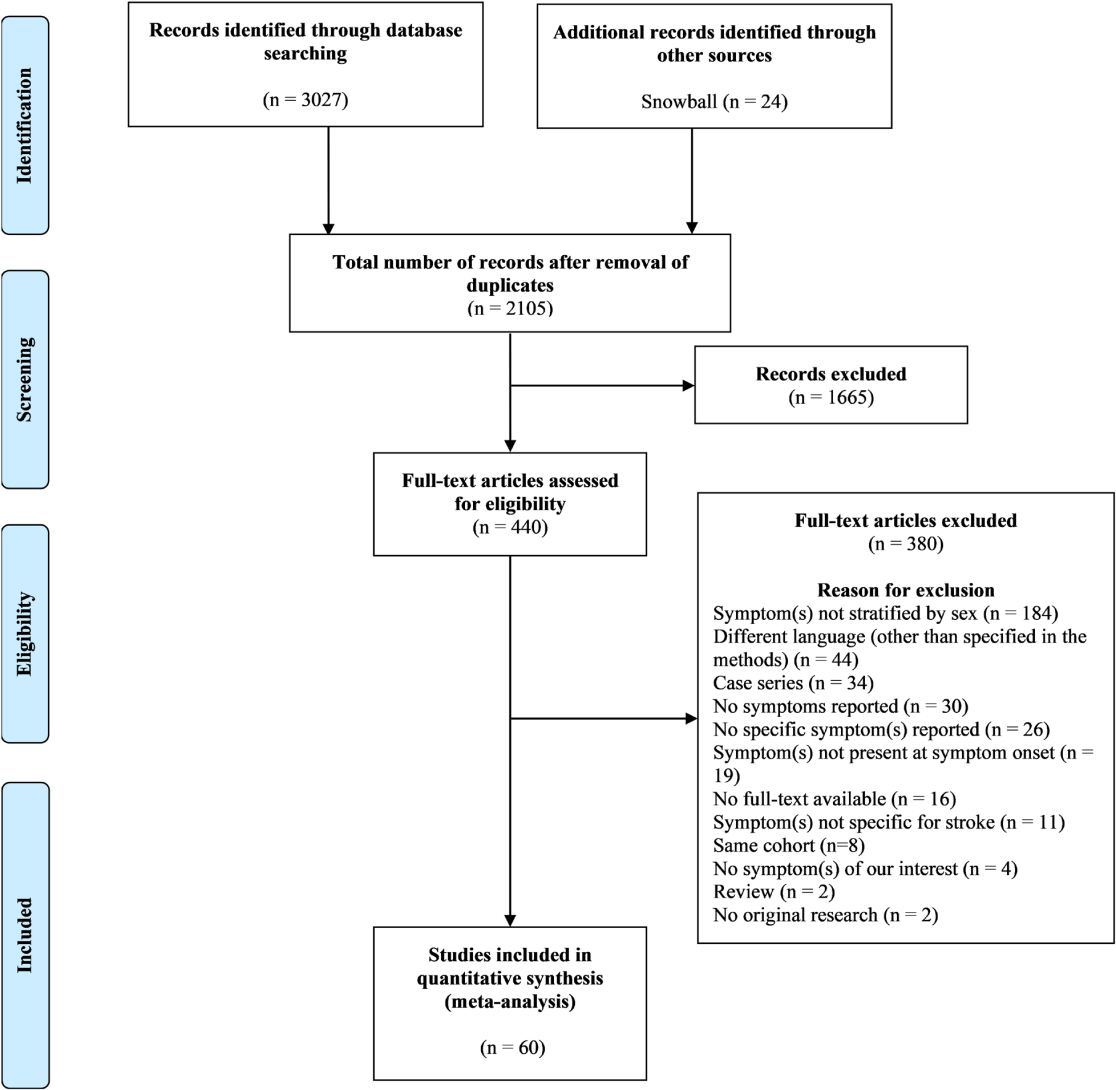
Preferred Reporting Items for Systematic Reviews and Meta-Analyses flowchart of inclusion of studies.

A total of 3051 publications were retrieved of which 60 studies were included (Figure 1).^3–7,13,17–70^ Study characteristics are summarized in Table S1. An overview of reported symptoms in included studies is given in Table S2. The 60 studies^3–7,13,17–70^ included a total of 582 844 patients (50% women). The median age was 74 years for women (interquartile range, 69–75) and 69 years for men (interquartile range, 64–70). Eighteen studies included ischemic strokes with a total of 51 824 patients (49% women),^5,7,19,22,23,27,29,30,32,40,41,49,52,54,56,62,65,68^ 4 studies included TIAs with 8004 patients (50% women),^3,31,38,50^ 3 studies included both ischemic strokes and TIAs with 5130 patients (45% women),^6,51,63^ and 3 studies included 842 patients (46% women) with ICH without data available on possible inclusion of SAH.^20,48,53^ Twenty-four^7,17,19,20,24,27,31,36,38,43,45,48,49,51,53,54,58,59,63,65,66,68,69,71^ of these 60 studies (68 958 patients) reported on nonfocal symptoms. Fourteen studies^25,26,29,30,32,35,37,39–41,46,47,61,64^ (18 299 patients) assessed focal symptoms. Twenty-two studies reported on both nonfocal and focal symptoms (496 187 patients).^3–6,13,18,21–23,28,33,34,42,44,50,52,55,56,60,62,67,70^ Thirty-three studies (56%) were multicenter cohorts.^3–5,7,13,17,19,20,22–25,27,28,33,34,36,39,42,46,48–51,55,56,58–60,63,65,66,69^ The total number of patients per study ranged from 59 to 398 798.

### Risk-of-Bias Assessment of Included Studies

Most studies contained 1 or 2 sources of bias, and no study fulfilled our criteria of high methodological quality. Therefore, the outcomes of our risk-of-bias assessment were not suitable to perform a subgroup analysis with studies containing low or relatively low risk of bias.

Fifty-one studies confirmed stroke through clinical evaluation and neuroimaging and were classified as having low risk of bias.^3,5–7,13,17–23,25,27–36,39–49,52–54,56–61,63–70^ Other articles assessed stroke through the *International Classification of Diseases*, *Tenth Revision*, codes (n=2)^4,50^ and medical records (n=5).^24,26,38,55,62^

Only 2 of the 60 studies provided both crude and age-adjusted comparisons between men and women.^36,62^ In total, 31 studies included all subtypes of stroke and imposed no selection criteria for their patient population and, therefore, had a low risk of bias.^4,13,17,18,20,24,25,28,33–37,39,41–43,45–47,55,57–61,64,66,67,69,70^ Moreover, 2 studies that included hemorrhagic stroke did not make a clear distinction between ICH and SAH.^48,53^ The remaining studies were classified as having high risk of bias. The results of the risk-of-bias assessment and the funnel plots are presented in Table S3 and Figure S8.

### Meta-Analysis for Any Type of Stroke

**Figure 2.**
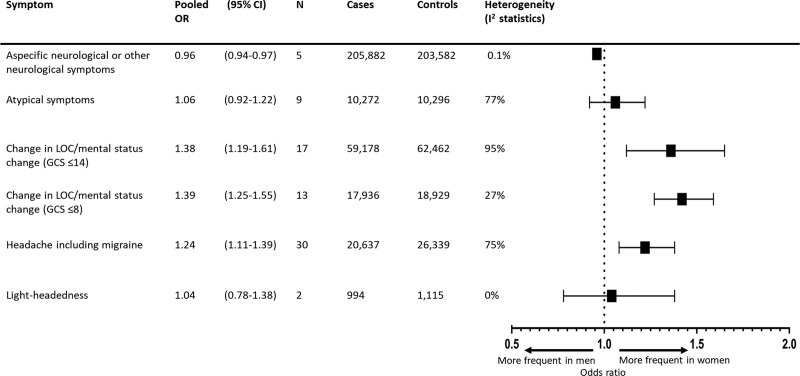
**Summary forest plot for nonfocal symptoms in patients with any type of stroke.** Cases: total number of women; controls: total number of men. GCS indicates Glasgow Coma Scale; LOC, level of consciousness; and OR, odds ratio.

The overall pooled OR of occurrence of nonfocal symptoms in women versus men was 1.24 (95% CI, 1.16–1.33) with a summary incidence of 27% for men versus 31% (95% CI, 30%–33%) for women and considerable heterogeneity (I^2^=91.9%). Headache including migraine (OR, 1.24 [95% CI, 1.11–1.39]; summary incidence: 16% for men versus 19% [95% CI, 17%–21%] for women; I^2^=75.2%; 30 studies; 47 254 patients), minor change in level of consciousness or mental status change (GCS score, ≤14; OR, 1.38 [95% CI, 1.19–1.61]; summary incidence: 17% for men versus 22% [95% CI, 20%–25%] for women; I^2^=95.0%; 17 studies; 122 465 patients), and coma or stupor (GCS score, ≤8; OR, 1.39 [95% CI, 1.25–1.55]; summary incidence: 6% for men versus 8% [95% CI, 7%–9%] for women; I^2^=27.0%; 13 studies; 37 196 patients) occurred more frequently in women compared with men. However, aspecific neurological or other neurological symptoms occurred less frequently in women (OR, 0.96 [95% CI, 0.94–0.97]; summary incidence: 32% for men versus 31% [95% CI, 31%–31%] for women; I^2^=0.1%; 5 studies; 409 464 patients; Figure 2; Figure S1). Because 1 study with risk of bias on the domain validation of diagnosis had a very large sample size (398 798 patients; 68% of the total number of patients), we chose to assess impact of this study on the association between aspecific neurological or other neurological symptoms and female sex.^55^ A post hoc sensitivity analysis was, therefore, performed by excluding this study, which showed no significant differences between women and men in occurrence of aspecific neurological or other neurological symptoms (OR, 0.95 [95% CI, 0.84–1.07]; I^2^=8.9%; 4 studies; 10 666 patients; Figure S2).

**Figure 3.**
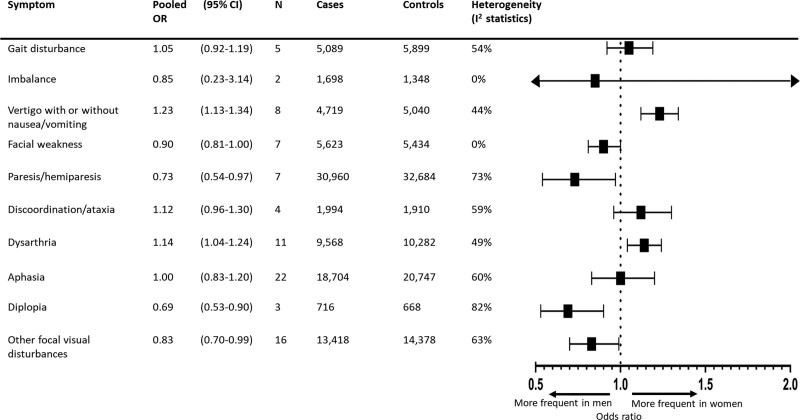
**Summary forest plot for focal symptoms in patients with any type of stroke.** Cases: total number of women; controls: total number of men. OR indicates odds ratio.

The overall pooled estimate of all focal symptoms was not associated with sex (OR, 1.03 [95% CI, 0.97–1.09]; summary incidence: 40% for men versus 41% [95% CI, 39%–42%] for women), but there was considerable heterogeneity (I^2^=78.8%). However, dysarthria (OR, 1.14 [95% CI, 1.04–1.24]; summary incidence: 37% for men versus 40% [95% CI, 38%–42%] for women; I^2^=48.6%; 11 studies; 20 385 patients) and vertigo with or without nausea/vomiting (OR, 1.23 [95% CI, 1.13–1.34]; summary incidence: 19% for men versus 22% [95% CI, 21%–24%] for women; I^2^=44.0%; 8 studies; 9759 patients) occurred significantly more frequently in women. In contrast, the focal symptoms paresis/hemiparesis (OR, 0.73 [95% CI, 0.54–0.97]; summary incidence: 73% for men versus 66% [95% CI, 59%–72%] for women; I^2^=72.6%; 7 studies; 63 605 patients), diplopia (OR, 0.69 [95% CI, 0.53–0.90]; summary incidence: 5% for men versus 4% [95% CI, 3%–5%] for women; I^2^=81.8%; 3 studies; 1384 patients), and other focal visual disturbances (OR, 0.83 [95% CI, 0.70–0.99]; summary incidence: 16% for men versus 14% [95% CI, 12%–16%] for women; I^2^=62.8%; 16 studies; 27 796 patients) occurred significantly less frequently in women compared with men (Figure 3; Figure S3).

### Ischemic Stroke (Non-TIA)

For a subgroup analysis for sex differences in ischemic stroke, we included 18 studies with ischemic stroke only^5,7,19,22,23,27,29,30,32,40,41,49,52,54,56,62,65,68^ and 3 studies that provided separate analyses for ischemic and hemorrhagic stroke.^17,39,44^ In total, 53 226 ischemic stroke patients were included.

Most of the effect estimates (including headache with or without migraine) failed to reach significance. However, the association between female sex and change in level of consciousness/mental status change (GCS score ≤8: OR, 1.50 [95% CI, 1.20–1.88]; I^2^=34.1%; n=4 studies and GCS score ≤14: OR, 1.38 [95% CI, 1.27–1.50]; I^2^=29.3%; n=4 studies) remained statistically significant. The results of the subgroup analysis are provided in Figure S4 (nonfocal symptoms) and Figure S5 (focal symptoms). Heterogeneity of the overall pooled analysis in the ischemic stroke subgroup decreased from 93.6% to 85.0% for nonfocal and from 87.9% to 79.0% for focal symptoms.

### Transient Ischemic Attack

In patients with TIA, we were only able to conduct a subgroup meta-analysis for headache. Headache was reported by all the studies that reported exclusively on a TIA population.^3,31,38,50^ The OR of headache occurrence in women compared with men was 1.42 ([95% CI, 1.27–1.58] I^2^=0.0%; 4 studies; 8004 patients; Figure S6).

### Subgroup Analysis for ICH or SAH

Due to the limited number of studies available on ICH, subgroup analysis for ICH was only possible for the symptom headache. Headache occurrence by ICH was reported by 5 studies^17,20,44,48,53^ with 1096 patients in total. SAH was reported to be excluded in 2 studies^17,20^ and included in another study.^44^ It was not clear whether the remaining 2 studies^48,53^ analyzed SAH as a separate entity and not in combination with ICH.

The subgroup analysis showed no statistical differences in headache occurrence by women and men (OR, 1.27 [95% CI, 0.80–2.02]; I^2^=67.6%; n=5 studies; Figure S7). No studies were available that reported exclusively on SAH.

## Discussion

In our systematic review and meta-analysis, the occurrence of nonfocal symptoms in women with stroke was higher than in men. More frequently occurring nonfocal symptoms were changes in level of consciousness/mental status and headache including migraine. In addition, significant sex differences were found for certain focal symptoms. Dysarthria and vertigo with or without nausea/vomiting were more common in women, whereas paresis/hemiparesis, diplopia, and other focal visual disturbances were more common in men. Significant heterogeneity, however, limits the reliability of these results. The subgroup analysis of patients with ischemic stroke resulted in low-to-moderate heterogeneity in several subgroups of symptoms and showed that most of the effect estimates were no longer statistically significant compared with the main analysis possibly because of loss of power. Although the association between headache and female sex disappeared, the association with change in level of consciousness was still apparent.

Four previously published reviews on stroke in women and sex differences in the evaluation and treatment of acute ischemic stroke also concluded that a higher proportion of women presented with nonfocal symptoms. In these studies, however, no formal meta-analyses were performed.^2,11,72,73^ Several individual studies have reported comparable incidences of nonfocal symptoms in men and women.^3,42,50,61^ Discrepancies between these individual studies and the reviews and our meta-analysis may be explained by the limited number of patients and various sources of potential bias in the individual studies. In contrast to the subgroup analysis of our meta-analysis, a recently published review based on 11 studies on stroke-related headache reported a higher incidence of headache during or after ischemic stroke in women (OR, 1.25 [95% CI, 1.07–1.46]).^74^ Differences in inclusion criteria most likely explain the difference with our results. In our subgroup analysis, we excluded studies that combined TIA and ischemic stroke and studies that did not enroll participants consecutively. In addition, because of our extensive literature search covering 5 databases and cross-reference check of included articles and relevant reviews, we were able to include 4 studies that were not identified by the previous systematic review.^5,22,44,62^

To interpret our results, several methodological challenges must be considered. First, significant heterogeneity hampered interpretation of the pooled effect estimates. The heterogeneity may be explained by characteristics that vary between studies, such as inclusion of heterogeneous study populations in terms of patient and stroke characteristics, study designs and data collection methods, definition of stroke end points, presence of several sources of bias, and differences in adjustment for confounders. In our subgroup analysis for ischemic stroke, the heterogeneity of pooled effect estimates was indeed substantially reduced for several symptoms, indicating that stroke subtype was an important contributor to heterogeneity. Second, adjustment for confounding factors was performed in a minority of the included studies. Only 2 studies adjusted for age, and after adjustment, several symptoms did not significantly differ from men.^36,62^ We, therefore, were only able to pool the unadjusted effect estimates. However, median age difference between women and men was on average limited (±5 years). In addition, our version of the Newcastle-Ottawa Scale was not validated.

Third, the majority of the studies contained at least 2 sources of bias, and no study fulfilled our criteria of high methodological quality. Fourth, although we included 60 studies for this meta-analysis, for several symptoms not enough studies were available to draw robust conclusions. Fifth, examination of patients with stroke varied considerably among studies. From the studies that reported the timing of symptom assessment, 5 studies examined stroke patients admitted within 72 hours after the onset of stroke symptoms,^3,7,21,52,63^ 3 other studies within 7 days after onset,^39,56,71^ and 1 study retrieved information on clinical presentation within 14 days from onset.^22^ The timing was unknown in the remaining studies. Sixth, some of the studies reported symptoms combined or defined symptom categories that could not be grouped in our symptom classification system. Thus, misclassification of several symptoms might have occurred since several symptom categories that were derived from the studies contained considerable overlap in definition. Seventh, asymmetry was observed in the funnel plot for studies reporting on aphasia, minor change in level of consciousness/mental status change, and coma or stupor possibly indicating publication bias. Furthermore, recall bias regarding presenting symptoms may have resulted in underestimation of the symptom occurrences. Moreover, selection bias attributable to the use of stroke-specific inclusion criteria may have influenced the external validity of the results. In addition, our meta-analysis is subject to within-study reporting bias: studies meeting selection criteria are more likely to contain significant results, as opposed to excluded or (non)published studies that could have reported appropriate, nonsignificant data. This may also explain a substantial proportion of the observed heterogeneity.

Eight, we were unable to make a distinction between ICH and SAH in our analyses. From our included studies, 24 studies included a study population with ischemic stroke or TIA only and 7 studies included all stroke subtypes except SAH. None of our studies reported exclusively on SAH. However, SAH is a relatively small contributor to overall stroke (around 5%).^75^ We, therefore, expect the influence of SAH in overall stroke or hemorrhagic stroke specifically to be small and consider it unlikely that the inclusion of SAH has importantly influenced our conclusion. It is remarkable that no studies on sex differences in clinical presentation of SAH exist. Given the importance of knowledge about sex differences in hemorrhagic stroke subtypes for daily clinical care, we recommend this should be further investigated in future studies.

The strengths of our systematic review and meta-analysis include the large number of included studies using all known contemporary data from almost 600 000 patients, the subgroup analysis by stroke type to assess impact on effect estimates and heterogeneity, and the evaluation of a wide variety of nonfocal and focal symptoms.

We have performed a sensitivity analysis, performed subgroup analyses by stroke type, and produced a table describing the risk of bias of the included studies to help explain heterogeneity and give the reader information to interpret the results given the limitations. Because of the substantial amount of limitations, our findings should be interpretated cautiously.

Several hypotheses exist for mechanisms underlying possible sex differences in stroke symptomatology. First, cardioembolic stroke and SAH occur more frequently in women.^61,76^ Second, sex differences in neurobiology should be considered. Women may, for example, be more sensitive to peri-infarction depolarizations that may lead to headache.^77^ Third, women tend to be older at stroke onset and have more comorbidities.^78^ The increased prevalence of dementia, psychosocial stressors, and depression among women could impact stroke presentation.^11^ Fourth, one study reported that women are more likely to be diagnosed with a stroke mimic such as migraine, seizure, or other psychiatric disorders, which could point toward caregivers’ biases toward patients’ sex.^3^ In addition, there are sex aspects on perceiving and acting on stroke symptoms; while women generally have a better knowledge of stroke symptoms, men are more likely to call an ambulance for themselves.^79^

Until more high-quality prospective cohort studies become available, we feel it is important that physicians are at least aware of the possible sex differences in presentation of stroke, especially for nonfocal symptoms such as headache and mental status changes. This awareness might be particularly important in women with acute onset of nonfocal symptoms without obvious focal symptoms of stroke.

## Conclusions

We found that women have a significant higher odds of presenting with nonfocal symptoms during acute stroke compared with men. Furthermore, significant sex differences were observed regarding focal stroke symptomatology. However, given the heterogeneity and poor methodological quality of our included studies, our findings should be considered as hypothesis generating. Additional research is required for more conclusive results. Future prospective cohort studies should be specifically designed to assess sex differences; should take confounding factors into account and adjust for at least age, stroke subtype, and comorbidity; and should report presenting nonfocal and focal symptoms by stroke subtype, timing, and location. Finally, studies need to assess the association between presenting symptoms and clinical diagnosis and should compare the symptoms of women and men with and without confirmed stroke to improve the diagnosis of stroke in clinical practice.

## Article Information

### Sources of Funding

Dr Wermer was supported by a personal grant from ZonMw (VIDI 91717337 and Aspasia). Dr van Os was supported by a personal Dekker Junior Clinical Scientist Grant from the Dutch Heart Foundation (2018T082).

### Disclosures

None.

### Supplemental Material

Supplemental Search Strategy

Detailed Search Query

Supplemental Methods

Tables S1–S3

Figures S1–S8

## Supplementary Material


