# Supplementary material for: Sex Differences in Presentation of Stroke: A Systematic Review and Meta-Analysis
Source: Stroke. 2021 Dec 14;53(2):345–54. doi: 10.1161/STROKEAHA.120.034040 (PMC8785516; doi:10.1161/STROKEAHA.120.034040)

SUPPLEMENTAL MATERIAL

Sex differences in presentation of stroke: a systematic review and meta-analysis.

Supplement search strategy

Detailed search query

Supplement Method. Protocol - risk of bias assessment according to the Newcastle-Ottawa Scale (customized version)

Supplement Table I. Characteristics of included studies

Supplement Table II. Reported symptoms in included studies

Supplement Table III. Risk of bias assessment of included studies

Supplement Figure I. Forest plot of non-focal symptoms

Supplement Figure II. Sensitivity analysis of nonspecific neurological or other neurological symptoms only (Mochari-Greenberger et al. excluded)

Supplement Figure III. Forest plot of focal symptoms

Supplement Figure IV. Forest plot of the subgroup analysis for non-focal symptoms in patients with ischemic stroke

Supplement Figure V. Forest plot of the subgroup analysis for focal symptoms in patients with ischemic stroke

Supplement Figure VI. Forest plot of the subgroup analysis for headache in patients with a TIA

Supplement Figure VII. Forest plot of the subgroup analysis for headache in patients with ICH or SAH

Supplement Figure VIII. Publication bias funnel plots

Supplement search strategy

We designed the search strategy in collaboration with an expert medical librarian. Search terms were formulated related to: 1) “stroke”, with distinction between ischemic and hemorrhagic stroke (including both subarachnoid hemorrhage [SAH] and intracerebral hemorrhage [ICH]) and TIA, 2) “sex” and “sex differences”, and 3) specific non-focal and focal symptoms. In addition, search terms were used to exclude case reports and reviews. We also searched reference lists from the included studies and relevant review articles to identify further relevant articles. This method of cross-checking was continued until no further relevant studies were found. No restrictions for publication dates were imposed.

Detailed search query

I. PubMed

(Final search run on 27-05-2020; 1384 items)

((("Stroke"[majr] OR "Stroke"[ti] OR "strokes"[ti] OR "CVA"[ti] OR "CVAs"[ti] OR "Cerebrovascular Accident"[ti] OR "Cerebrovascular Accidents"[ti] OR "Cerebrovascular Insult"[ti] OR "Cerebrovascular Insults"[ti] OR "CVI"[ti] OR "CVIs"[ti] OR "Brain Attack"[ti] OR "Brain Attacks"[ti] OR Brain Infarct*[ti] OR "Brain Infarction"[majr] OR "Cerebrovascular Apoplexy"[ti] OR "Apoplexy"[ti] OR "Brain Vascular Accident"[ti] OR "Brain Vascular Accidents"[ti] OR "Cerebrovascular Stroke"[ti] OR "Cerebrovascular Strokes"[ti] OR "Cerebral Stroke"[ti] OR "Cerebral Strokes"[ti] OR "Acute Stroke"[ti] OR "Acute Strokes"[ti] OR "Acute Cerebrovascular Accident"[ti] OR "Acute Cerebrovascular Accidents"[ti] OR "Cerebrovascular Event"[ti] OR "Cerebrovascular Events"[ti] OR "Cerebrovascular Attack"[ti] OR "Cerebrovascular Attacks"[ti] OR "Cerebral Infarction"[majr] OR Cerebral infarct*[ti] OR "Brain ischemia"[majr] OR "Brain ischemia"[ti] OR "Brain ischaemia"[ti] OR "Cerebral Ischemia"[ti] OR "Cerebral Ischaemia"[ti] OR "Cerebral Hemorrhage"[majr] OR Cerebral Hemorrhag*[ti] OR Cerebral Haemorrhag*[ti] OR Intracerebral Hemorrhag*[ti] OR Brain Hemorrhag*[ti] OR Brain Haemorrhag*[ti] OR Intracerebral Haemorrhag*[ti] OR "Neurologic Event"[ti] OR "Neurologic Events"[ti] OR "Ischemic Attack, Transient"[majr] OR "transient ischemic attack"[ti] OR "transient ischemic attack"[ti] OR "transient ischaemic attack"[ti] OR "transient ischaemic attacks"[ti] OR "TIA"[ti] OR "TIAs"[ti] OR "transient brainstem ischemia"[ti] OR "transient brainstem ischaemia"[ti] OR "transient brain stem ischemia"[ti] OR "transient brain stem ischaemia"[ti] OR "transient brain ischemia"[ti] OR "transient brain ischaemia"[ti] OR "transient cerebral ischemia"[ti] OR "transient cerebral ischaemia"[ti] OR "transient cerebral ischemic"[ti] OR "transient cerebral ischaemic"[ti] OR ("transient"[ti] AND ("brain"[ti] OR "cerebral"[ti]) AND (ischemi*[ti] OR ischaem*[ti]))) AND ("Sex"[Mesh] OR "Gender"[tw] OR "Sex"[tiab] OR "Sex Characteristics"[Mesh] OR Sex Characteristic*[tw] OR Sex Difference*[tw] OR Gender Difference*[tw] OR "Sex Based"[tw] OR Sex Dimorphism*[tw] OR Sexual Dimorphism*[tw] OR (("Women"[mesh] OR "Women"[tw] OR "Woman"[tw] OR "Female"[tiab]) AND ("Men"[mesh] OR "Men"[tw] OR "Man"[tw] OR "Male"[tiab]))) AND ("Pain"[mesh] OR "pain"[tw] OR "facial pain"[tw] OR "hemi-body pain"[mesh] OR "Confusion"[mesh:NoExp] OR "disorientation"[tw] OR disorient*[tw] OR "confusion"[tw] OR confus*[tw] OR "Unconsciousness"[mesh] OR "Unconsciousness"[tw] OR "loss of consciousness"[tw] OR "Consciousness Disorders"[mesh] OR "decreased consciousness"[tw] OR "Dizziness"[mesh] OR "Dizziness"[tw] OR "lightheadedness" [tw] OR "headache"[tw] OR headach*[tw] OR "Nausea"[mesh:NoExp] OR "Nausea"[tw] OR "nonfocal weakness"[tw] OR "non-focal weakness"[tw] OR "nonfocal symptom"[tw] OR "non-focal symptom"[tw] OR "nonfocal symptoms"[tw] OR "non-focal symptoms"[tw] OR "non-neurological symptoms"[tw] OR "Chest Pain"[mesh] OR "chest pain"[tw] OR "palpitations"[tw] OR "palpitation"[tw] OR "Dyspnea"[mesh] OR "Dyspnea"[tw] OR "shortness of breath" [tw] OR "hemi-body numbness"[tw] OR "hemibody numbness"[tw] OR "numbness"[tw] OR "Hypesthesia"[Mesh] OR hypesthesi*[tw] OR "Diplopia"[mesh] OR "diplopia"[tw] OR

"Vision Disorders"[mesh:noexp] OR "Vision Disorders"[tw] OR "Vision Disorder"[tw] OR "visual disturbances"[tw] OR "visual disturbance"[tw] OR "vision disturbances"[tw] OR "vision disturbance"[tw] OR "Blindness"[mesh] OR "Blindness"[tw] OR "Hemianopsia"[mesh] OR "hemianopsia"[tw] OR "Aphasia"[mesh] OR "aphasia"[tw] OR "Dysarthria"[mesh] OR "dysarthria"[tw] OR "discoordination"[tw] OR "Ataxia"[mesh] OR "ataxia"[tw] OR "hemiparesis"[tw] OR "haemiparesis"[tw] OR "Paresis"[mesh] OR "Paresis"[tw] OR "facial weakness"[tw] OR "Facial Paralysis"[mesh] OR "Facial Paralysis"[tw] OR "Vertigo"[mesh] OR "vertigo"[tw] OR "Change in vision"[tw] OR "vision change"[tw] OR "vision changes"[tw] OR "Vision problem"[tw] OR "Hemianopia"[tw] OR "Blurred vision"[tw] OR "Blurry Vision"[tw] OR "Decreased Vision"[tw] OR "Double Vision"[tw] OR "Vision Alteration"[tw] OR "Visual Deficit"[tw] OR "Visual Deficits"[tw] OR "Visual Field Defect"[tw] OR "Visual Field Defects"[tw] OR "Visual Field Loss"[tw] OR "Loss of Vision"[tw] OR "vision loss"[tw] OR "Monocular Blindness"[tw] OR "Unconsciousness"[Mesh] OR unconscious*[tw] OR "Consciousness"[Mesh] OR conscious*[tw] OR "drowsy"[tw] OR drows*[tw] OR "Aphasia"[mesh] OR aphasi*[tw] OR "visual field disturbances"[tw] OR "visual field disturbance"[tw] OR "Vision Disorders"[Mesh] OR "dysphagia"[tw] OR "Deglutition Disorders"[Mesh] OR "clinical course"[tw])) OR (("Stroke"[majr] OR "Stroke"[ti] OR "strokes"[ti] OR "CVA"[ti] OR "CVAs"[ti] OR "Cerebrovascular Accident"[ti] OR "Cerebrovascular Accidents"[ti] OR "Cerebrovascular Insult"[ti] OR "Cerebrovascular Insults"[ti] OR "CVI"[ti] OR "CVIs"[ti] OR "Brain Attack"[ti] OR "Brain Attacks"[ti] OR Brain Infarct*[ti] OR "Brain Infarction"[majr] OR "Cerebrovascular Apoplexy"[ti] OR "Apoplexy"[ti] OR "Brain Vascular Accident"[ti] OR "Brain Vascular Accidents"[ti] OR "Cerebrovascular Stroke"[ti] OR "Cerebrovascular Strokes"[ti] OR "Cerebral Stroke"[ti] OR "Cerebral Strokes"[ti] OR "Acute Stroke"[ti] OR "Acute Strokes"[ti] OR "Acute Cerebrovascular Accident"[ti] OR "Acute Cerebrovascular Accidents"[ti] OR "Cerebrovascular Event"[ti] OR "Cerebrovascular Events"[ti] OR "Cerebrovascular Attack"[ti] OR "Cerebrovascular Attacks"[ti] OR "Cerebral Infarction"[majr] OR Cerebral infarct*[ti] OR "Brain ischemia"[majr] OR "Brain ischemia"[ti] OR "Brain ischaemia"[ti] OR "Cerebral Ischemia"[ti] OR "Cerebral Ischaemia"[ti] OR "Cerebral Hemorrhage"[majr] OR Cerebral Hemorrhag*[ti] OR Cerebral Haemorrhag*[ti] OR Intracerebral Hemorrhag*[ti] OR Brain Hemorrhag*[ti] OR Brain Haemorrhag*[ti] OR Intracerebral Haemorrhag*[ti] OR "Neurologic Event"[ti] OR "Neurologic Events"[ti] OR "Ischemic Attack, Transient"[majr] OR "transient ischemic attack"[ti] OR "transient ischemic attack"[ti] OR "transient ischaemic attack"[ti] OR "transient ischaemic attacks"[ti] OR "TIA"[ti] OR "TIAs"[ti] OR "transient brainstem ischemia"[ti] OR "transient brainstem ischaemia"[ti] OR "transient brain stem ischemia"[ti] OR "transient brain stem ischaemia"[ti] OR "transient brain ischemia"[ti] OR "transient brain ischaemia"[ti] OR "transient cerebral ischemia"[ti] OR "transient cerebral ischaemia"[ti] OR "transient cerebral ischemic"[ti] OR "transient cerebral ischaemic"[ti] OR ("transient"[ti] AND ("brain"[ti] OR "cerebral"[ti]) AND (ischemi*[ti] OR ischaem*[ti]))) AND ("Sex"[majr] OR "Gender"[ti] OR "Sex"[ti] OR "sexes"[ti] OR "Sex Characteristics"[majr] OR Sex Characteristic*[ti] OR Sex Difference*[ti] OR Gender Difference*[ti] OR "Sex Based"[ti] OR Sex Dimorphism*[ti] OR Sexual Dimorphism*[ti] OR (("Women"[majr] OR "Women"[ti] OR "Woman"[ti] OR "Female"[ti]) AND ("Men"[majr] OR "Men"[ti] OR "Man"[ti] OR

"Male"[ti]))) AND (differenc*[ti] OR disparit*[ti] OR sex differenc*[tiab] OR sex disparit*[tiab] OR gender differenc*[tiab] OR gender disparit*[tiab] OR "differences between men and women"[tw]) AND ("symptom"[tw] OR "symptoms"[tw]))) NOT (("Case Reports"[ptyp] OR "case report"[ti] OR "Review"[ptyp] OR "review"[ti]) NOT ("Clinical Study"[ptyp] OR "trial"[ti] OR "RCT"[ti]))

II. Embase
(Final search run on 27-05-2020; 693 items)

(((exp *"cerebrovascular accident"/ OR "Stroke".ti OR "strokes".ti OR "CVA".ti OR "CVAs".ti OR "Cerebrovascular Accident".ti OR "Cerebrovascular Accidents".ti OR "Cerebrovascular Insult".ti OR "Cerebrovascular Insults".ti OR "CVI".ti OR "CVIs".ti OR "Brain Attack".ti OR "Brain Attacks".ti OR "Brain Infarct*".ti OR exp *"Brain Infarction"/ OR "Cerebrovascular Apoplexy".ti OR "Apoplexy".ti OR "Brain Vascular Accident".ti OR "Brain Vascular Accidents".ti OR "Cerebrovascular Stroke".ti OR "Cerebrovascular Strokes".ti OR "Cerebral Stroke".ti OR "Cerebral Strokes".ti OR "Acute Stroke".ti OR "Acute Strokes".ti OR "Acute Cerebrovascular Accident".ti OR "Acute Cerebrovascular Accidents".ti OR "Cerebrovascular Event".ti OR "Cerebrovascular Events".ti OR "Cerebrovascular Attack".ti OR "Cerebrovascular Attacks".ti OR "Cerebral infarct*".ti OR exp *"Brain ischemia"/ OR "Brain ischemia".ti OR "Brain ischaemia".ti OR "Cerebral Ischemia".ti OR "Cerebral Ischaemia".ti OR exp *"Brain Hemorrhage"/ OR "Cerebral Hemorrhag*".ti OR "Cerebral Haemorrhag*".ti OR "Intracerebral Hemorrhag*".ti OR "Brain Hemorrhag*".ti OR "Brain Haemorrhag*".ti OR "Intracerebral Haemorrhag*".ti OR "Neurologic Event".ti OR "Neurologic Events".ti OR "Transient Ischemic Attack"/ OR "transient ischemic attack".ti OR "transient ischemic attack".ti OR "transient ischaemic attack".ti OR "transient ischaemic attacks".ti OR "TIA".ti OR "TIAs".ti OR "transient brainstem ischemia".ti OR "transient brainstem ischaemia".ti OR "transient brain stem ischemia".ti OR "transient brain stem ischaemia".ti OR "transient brain ischemia".ti OR "transient brain ischaemia".ti OR "transient cerebral ischemia".ti OR "transient cerebral ischaemia".ti OR "transient cerebral ischemic".ti OR "transient cerebral ischaemic".ti OR ("transient".ti ADJ3 ("brain".ti OR "cerebral".ti) ADJ3 (ischemi*.ti OR ischaem*.ti))) AND (*"Sex"/ OR "Gender".ti,ab OR "Sex".ti,ab OR exp *"Sexual Characteristics"/ OR "Sex Characteristic*".ti,ab OR "Sex Difference*".ti,ab OR "Gender Difference*".ti,ab OR "Sex Based".ti,ab OR "Sex Dimorphism*".ti,ab OR "Sexual Dimorphism*".ti,ab OR (("Women".ti,ab OR "Woman".ti,ab OR "Female".ti,ab) AND ("Men".ti,ab OR "Man".ti,ab OR "Male".ti,ab))) AND (exp *"Pain"/ OR "pain".ti OR "facial pain".ti OR "Confusion"/ OR "disorientation".ti OR disorient*.ti OR "confusion".ti OR confus*.ti OR exp *"Unconsciousness"/ OR "Unconsciousness".ti OR "loss of consciousness".ti OR exp *"Consciousness Disorder"/ OR "decreased consciousness".ti OR "Dizziness"/ OR "Dizziness".ti OR "lightheadedness".ti OR exp *"headache"/ OR "headache".ti OR headach*.ti OR exp *"Nausea"/ OR "Nausea".ti OR "nonfocal weakness".ti OR "non-focal weakness".ti OR "nonfocal symptom".ti OR "non-focal symptom".ti OR "nonfocal symptoms".ti OR "non-focal symptoms".ti OR "non-neurological symptoms".ti OR "Thorax Pain"/ OR "chest pain".ti OR "palpitations".ti OR "palpitation".ti OR exp *"Dyspnea"/ OR "Dyspnea".ti OR "shortness of breath" .ti OR "hemi-body numbness".ti OR "hemibody numbness".ti OR "numbness".ti OR exp *"Hypesthesia"/ OR hypesthesi*.ti OR exp *"Diplopia"/ OR "diplopia".ti OR "Visual Disorder"/ OR "Vision Disorders".ti OR "Vision Disorder".ti OR "visual disturbances".ti OR "visual disturbance".ti OR "vision disturbances".ti OR "vision disturbance".ti OR exp *"Blindness"/ OR "Blindness".ti OR exp *"Hemianopsia"/ OR "hemianopsia".ti OR exp *"Aphasia"/ OR "aphasia".ti OR exp *"Dysarthria"/ OR "dysarthria".ti OR

"discoordination".ti OR exp *"Ataxia"/ OR "ataxia".ti OR "hemiparesis".ti OR "haemiparesis".ti OR exp *"Paresis"/ OR "Paresis".ti OR "facial weakness".ti OR exp *"Facial Paralysis"/ OR "Facial Paralysis".ti OR exp *"Vertigo"/ OR "vertigo".ti OR "Change in vision".ti OR "vision change".ti OR "vision changes".ti OR "Vision problem".ti OR "Hemianopia".ti OR "Blurred vision".ti OR "Blurry Vision".ti OR "Decreased Vision".ti OR "Double Vision".ti OR "Vision Alteration".ti OR "Visual Deficit".ti OR "Visual Deficits".ti OR "Visual Field Defect".ti OR "Visual Field Defects".ti OR "Visual Field Loss".ti OR "Loss of Vision".ti OR "vision loss".ti OR "Monocular Blindness".ti OR exp *"Consciousness"/ OR conscious*.ti OR "drowsy".ti OR drows*.ti OR "visual field disturbances".ti OR "visual field disturbance".ti OR "dysphagia".ti OR exp *"Dysphagia"/ OR "clinical course".ti)) OR ((exp *"cerebrovascular accident"/ OR "Stroke".ti OR "strokes".ti OR "CVA".ti OR "CVAs".ti OR "Cerebrovascular Accident".ti OR "Cerebrovascular Accidents".ti OR "Cerebrovascular Insult".ti OR "Cerebrovascular Insults".ti OR "CVI".ti OR "CVIs".ti OR "Brain Attack".ti OR "Brain Attacks".ti OR "Brain Infarct*".ti OR exp *"Brain Infarction"/ OR "Cerebrovascular Apoplexy".ti OR "Apoplexy".ti OR "Brain Vascular Accident".ti OR "Brain Vascular Accidents".ti OR "Cerebrovascular Stroke".ti OR "Cerebrovascular Strokes".ti OR "Cerebral Stroke".ti OR "Cerebral Strokes".ti OR "Acute Stroke".ti OR "Acute Strokes".ti OR "Acute Cerebrovascular Accident".ti OR "Acute Cerebrovascular Accidents".ti OR "Cerebrovascular Event".ti OR "Cerebrovascular Events".ti OR "Cerebrovascular Attack".ti OR "Cerebrovascular Attacks".ti OR "Cerebral infarct*".ti OR exp *"Brain ischemia"/ OR "Brain ischemia".ti OR "Brain ischaemia".ti OR "Cerebral Ischemia".ti OR "Cerebral Ischaemia".ti OR exp *"Brain Hemorrhage"/ OR "Cerebral Hemorrhag*".ti OR "Cerebral Haemorrhag*".ti OR "Intracerebral Hemorrhag*".ti OR "Brain Hemorrhag*".ti OR "Brain Haemorrhag*".ti OR "Intracerebral Haemorrhag*".ti OR "Neurologic Event".ti OR "Neurologic Events".ti OR "Transient Ischemic Attack"/ OR "transient ischemic attack".ti OR "transient ischemic attack".ti OR "transient ischaemic attack".ti OR "transient ischaemic attacks".ti OR "TIA".ti OR "TIAs".ti OR "transient brainstem ischemia".ti OR "transient brainstem ischaemia".ti OR "transient brain stem ischemia".ti OR "transient brain stem ischaemia".ti OR "transient brain ischemia".ti OR "transient brain ischaemia".ti OR "transient cerebral ischemia".ti OR "transient cerebral ischaemia".ti OR "transient cerebral ischemic".ti OR "transient cerebral ischaemic".ti OR ("transient".ti ADJ3 ("brain".ti OR "cerebral".ti) ADJ3 (ischemi*.ti OR ischaem*.ti))) AND (*"Sex"/ OR "Gender".ti OR "Sex".ti OR exp *"Sexual Characteristics"/ OR "Sex Characteristic*".ti OR "Sex Difference*".ti OR "Gender Difference*".ti OR "Sex Based".ti OR "Sex Dimorphism*".ti OR "Sexual Dimorphism*".ti OR (("Women".ti OR "Woman".ti OR "Female".ti) AND ("Men".ti OR "Man".ti OR "Male".ti))) AND (exp *"Pain"/ OR "pain".ti,ab OR "facial pain".ti,ab OR "Confusion"/ OR "disorientation".ti,ab OR disorient*.ti,ab OR "confusion".ti,ab OR confus*.ti,ab OR exp *"Unconsciousness"/ OR "Unconsciousness".ti,ab OR "loss of consciousness".ti,ab OR exp *"Consciousness Disorder"/ OR "decreased consciousness".ti,ab OR "Dizziness"/ OR "Dizziness".ti,ab OR "lightheadedness".ti,ab OR exp *"headache"/ OR "headache".ti,ab OR headach*.ti,ab OR exp *"Nausea"/ OR "Nausea".ti,ab OR "nonfocal weakness".ti,ab OR "non-focal weakness".ti,ab OR "nonfocal symptom".ti,ab OR "non-focal symptom".ti,ab OR "nonfocal symptoms".ti,ab OR "non-focal symptoms".ti,ab OR "non-neurological

symptoms".ti,ab OR "Thorax Pain"/ OR "chest pain".ti,ab OR "palpitations".ti,ab OR "palpitation".ti,ab OR exp *"Dyspnea"/ OR "Dyspnea".ti,ab OR "shortness of breath" .ti,ab OR "hemi-body numbness".ti,ab OR "hemibody numbness".ti,ab OR "numbness".ti,ab OR exp *"Hypesthesia"/ OR hypesthesi*.ti,ab OR exp *"Diplopia"/ OR "diplopia".ti,ab OR "Visual Disorder"/ OR "Vision Disorders".ti,ab OR "Vision Disorder".ti,ab OR "visual disturbances".ti,ab OR "visual disturbance".ti,ab OR "vision disturbances".ti,ab OR "vision disturbance".ti,ab OR exp *"Blindness"/ OR "Blindness".ti,ab OR exp *"Hemianopsia"/ OR "hemianopsia".ti,ab OR exp *"Aphasia"/ OR "aphasia".ti,ab OR exp *"Dysarthria"/ OR "dysarthria".ti,ab OR "discoordination".ti,ab OR exp *"Ataxia"/ OR "ataxia".ti,ab OR "hemiparesis".ti,ab OR "haemiparesis".ti,ab OR exp *"Paresis"/ OR "Paresis".ti,ab OR "facial weakness".ti,ab OR exp *"Facial Paralysis"/ OR "Facial Paralysis".ti,ab OR exp *"Vertigo"/ OR "vertigo".ti,ab OR "Change in vision".ti,ab OR "vision change".ti,ab OR "vision changes".ti,ab OR "Vision problem".ti,ab OR "Hemianopia".ti,ab OR "Blurred vision".ti,ab OR "Blurry Vision".ti,ab OR "Decreased Vision".ti,ab OR "Double Vision".ti,ab OR "Vision Alteration".ti,ab OR "Visual Deficit".ti,ab OR "Visual Deficits".ti,ab OR "Visual Field Defect".ti,ab OR "Visual Field Defects".ti,ab OR "Visual Field Loss".ti,ab OR "Loss of Vision".ti,ab OR "vision loss".ti,ab OR "Monocular Blindness".ti,ab OR exp *"Consciousness"/ OR conscious*.ti,ab OR "drowsy".ti,ab OR drows*.ti,ab OR "visual field disturbances".ti,ab OR "visual field disturbance".ti,ab OR "dysphagia".ti,ab OR exp *"Dysphagia"/ OR "clinical course".ti,ab)) OR ((exp *"Stroke"/ OR "Stroke".ti OR "strokes".ti OR "CVA".ti OR "CVAs".ti OR "Cerebrovascular Accident".ti OR "Cerebrovascular Accidents".ti OR "Cerebrovascular Insult".ti OR "Cerebrovascular Insults".ti OR "CVI".ti OR "CVIs".ti OR "Brain Attack".ti OR "Brain Attacks".ti OR "Brain Infarct*".ti OR exp *"Brain Infarction"/ OR "Cerebrovascular Apoplexy".ti OR "Apoplexy".ti OR "Brain Vascular Accident".ti OR "Brain Vascular Accidents".ti OR "Cerebrovascular Stroke".ti OR "Cerebrovascular Strokes".ti OR "Cerebral Stroke".ti OR "Cerebral Strokes".ti OR "Acute Stroke".ti OR "Acute Strokes".ti OR "Acute Cerebrovascular Accident".ti OR "Acute Cerebrovascular Accidents".ti OR "Cerebrovascular Event".ti OR "Cerebrovascular Events".ti OR "Cerebrovascular Attack".ti OR "Cerebrovascular Attacks".ti OR "Cerebral infarct*".ti OR exp *"Brain ischemia"/ OR "Brain ischemia".ti OR "Brain ischaemia".ti OR "Cerebral Ischemia".ti OR "Cerebral Ischaemia".ti OR exp *"Brain Hemorrhage"/ OR "Cerebral Hemorrhag*".ti OR "Cerebral Haemorrhag*".ti OR "Intracerebral Hemorrhag*".ti OR "Brain Hemorrhag*".ti OR "Brain Haemorrhag*".ti OR "Intracerebral Haemorrhag*".ti OR "Neurologic Event".ti OR "Neurologic Events".ti OR "Transient Ischemic Attack, Transient"/ OR "transient ischemic attack".ti OR "transient ischemic attack".ti OR "transient ischaemic attack".ti OR "transient ischaemic attacks".ti OR "TIA".ti OR "TIAs".ti OR "transient brainstem ischemia".ti OR "transient brainstem ischaemia".ti OR "transient brain stem ischemia".ti OR "transient brain stem ischaemia".ti OR "transient brain ischemia".ti OR "transient brain ischaemia".ti OR "transient cerebral ischemia".ti OR "transient cerebral ischaemia".ti OR "transient cerebral ischemic".ti OR "transient cerebral ischaemic".ti OR ("transient".ti ADJ3 ("brain".ti OR "cerebral".ti) ADJ3 (ischemi*.ti OR ischaem*.ti))) AND (*"Sex"/ OR "Gender".ti OR "Sex".ti OR "sexes".ti OR exp *"Sexual Characteristics"/ OR "Sex Characteristic*".ti OR "Sex Difference*".ti OR "Gender Difference*".ti OR "Sex

Based".ti OR "Sex Dimorphism*".ti OR "Sexual Dimorphism*".ti OR ((*"Female"/ OR "Women".ti OR "Woman".ti OR "Female".ti) AND (*"Male"/ OR "Men".ti OR "Man".ti OR "Male".ti))) AND (differenc*.ti OR disparit*.ti OR sex differenc*.ti,ab OR sex disparit*.ti,ab OR gender differenc*.ti,ab OR gender disparit*.ti,ab OR "differences between men and women".ti,ab) AND ("symptom".ti,ab OR "symptoms".ti,ab))) NOT (("Case Report"/ OR "case report".ti OR exp "Review"/ OR "review".ti) NOT ("Clinical Study"/ OR exp "Clinical Trial"/ OR "trial".ti OR "RCT".ti))

III. Web of Science

(Final search run on 27-05-2020; 288 items)

((ti=("cerebrovascular accident" OR "Stroke" OR "strokes" OR "CVA" OR "CVAs" OR "Cerebrovascular Accident" OR "Cerebrovascular Accidents" OR "Cerebrovascular Insult" OR "Cerebrovascular Insults" OR "CVI" OR "CVIs" OR "Brain Attack" OR "Brain Attacks" OR "Brain Infarct*" OR "Brain Infarction" OR "Cerebrovascular Apoplexy" OR "Apoplexy" OR "Brain Vascular Accident" OR "Brain Vascular Accidents" OR "Cerebrovascular Stroke" OR "Cerebrovascular Strokes" OR "Cerebral Stroke" OR "Cerebral Strokes" OR "Acute Stroke" OR "Acute Strokes" OR "Acute Cerebrovascular Accident" OR "Acute Cerebrovascular Accidents" OR "Cerebrovascular Event" OR "Cerebrovascular Events" OR "Cerebrovascular Attack" OR "Cerebrovascular Attacks" OR "Cerebral infarct*" OR "Brain ischemia" OR "Brain ischemia" OR "Brain ischaemia" OR "Cerebral Ischemia" OR "Cerebral Ischaemia" OR "Brain Hemorrhage" OR "Cerebral Hemorrhag*" OR "Cerebral Haemorrhag*" OR "Intracerebral Hemorrhag*" OR "Brain Hemorrhag*" OR "Brain Haemorrhag*" OR "Intracerebral Haemorrhag*" OR "Neurologic Event" OR "Neurologic Events" OR "Transient Ischemic Attack" OR "transient ischemic attack" OR "transient ischemic attack" OR "transient ischaemic attack" OR "transient ischaemic attacks" OR "TIA" OR "TIAs" OR "transient brainstem ischemia" OR "transient brainstem ischaemia" OR "transient brain stem ischemia" OR "transient brain stem ischaemia" OR "transient brain ischemia" OR "transient brain ischaemia" OR "transient cerebral ischemia" OR "transient cerebral ischaemia" OR "transient cerebral ischemic" OR "transient cerebral ischaemic" OR ("transient" AND ("brain" OR "cerebral") AND (ischemi* OR ischaem*))) AND ts=("Sex" OR "Gender" OR "Sex" OR "Sexual Characteristics" OR "Sex Characteristic*" OR "Sex Difference*" OR "Gender Difference*" OR "Sex Based" OR "Sex Dimorphism*" OR "Sexual Dimorphism*" OR (("Female" OR "Women" OR "Woman" OR "Female") AND ("Male" OR "Men" OR "Man" OR "Male"))) AND ti=("Pain" OR "pain" OR "facial pain" OR "Confusion" OR "disorientation" OR disorient* OR "confusion" OR confus* OR "Unconsciousness" OR "Unconsciousness" OR "loss of consciousness" OR "Consciousness Disorder" OR "decreased consciousness" OR "Dizziness" OR "Dizziness" OR "lightheadedness" OR "headache" OR headach* OR "Nausea" OR "Nausea" OR "nonfocal weakness" OR "non-focal weakness" OR "nonfocal symptom" OR "non-focal symptom" OR "nonfocal symptoms" OR "non-focal symptoms" OR "non-neurological symptoms" OR "Thorax Pain" OR "chest pain" OR "palpitations" OR "palpitation" OR "Dyspnea" OR "Dyspnea" OR "shortness of breath" OR "hemi-body numbness" OR "hemibody numbness" OR "numbness" OR "Hypesthesia" OR hypesthesi* OR "Diplopia" OR "diplopia" OR "Visual Disorder" OR "Vision Disorders" OR "Vision Disorder" OR "visual disturbances" OR "visual disturbance" OR "vision disturbances" OR "vision disturbance" OR "Blindness" OR "Blindness" OR "Hemianopsia" OR "hemianopsia" OR "Aphasia" OR "aphasia" OR "Dysarthria" OR "dysarthria" OR "discoordination" OR "Ataxia" OR "ataxia" OR "hemiparesis" OR "haemiparesis" OR "Paresis" OR "Paresis" OR "facial weakness" OR "Facial Paralysis" OR "Facial Paralysis" OR "Vertigo" OR "vertigo" OR "Change in vision" OR "vision change" OR "vision changes" OR "Vision problem" OR "Hemianopia" OR "Blurred vision" OR "Blurry Vision" OR "Decreased Vision" OR

"Double Vision" OR "Vision Alteration" OR "Visual Deficit" OR "Visual Deficits" OR "Visual Field Defect" OR "Visual Field Defects" OR "Visual Field Loss" OR "Loss of Vision" OR "vision loss" OR "Monocular Blindness" OR "Consciousness" OR conscious* OR "drowsy" OR drows* OR "visual field disturbances" OR "visual field disturbance" OR "dysphagia" OR "Dysphagia" OR "clinical course")) OR (ti=("cerebrovascular accident" OR "Stroke" OR "strokes" OR "CVA" OR "CVAs" OR "Cerebrovascular Accident" OR "Cerebrovascular Accidents" OR "Cerebrovascular Insult" OR "Cerebrovascular Insults" OR "CVI" OR "CVIs" OR "Brain Attack" OR "Brain Attacks" OR "Brain Infarct*" OR "Brain Infarction" OR "Cerebrovascular Apoplexy" OR "Apoplexy" OR "Brain Vascular Accident" OR "Brain Vascular Accidents" OR "Cerebrovascular Stroke" OR "Cerebrovascular Strokes" OR "Cerebral Stroke" OR "Cerebral Strokes" OR "Acute Stroke" OR "Acute Strokes" OR "Acute Cerebrovascular Accident" OR "Acute Cerebrovascular Accidents" OR "Cerebrovascular Event" OR "Cerebrovascular Events" OR "Cerebrovascular Attack" OR "Cerebrovascular Attacks" OR "Cerebral infarct*" OR "Brain ischemia" OR "Brain ischemia" OR "Brain ischaemia" OR "Cerebral Ischemia" OR "Cerebral Ischaemia" OR "Brain Hemorrhage" OR "Cerebral Hemorrhag*" OR "Cerebral Haemorrhag*" OR "Intracerebral Hemorrhag*" OR "Brain Hemorrhag*" OR "Brain Haemorrhag*" OR "Intracerebral Haemorrhag*" OR "Neurologic Event" OR "Neurologic Events" OR "Transient Ischemic Attack" OR "transient ischemic attack" OR "transient ischemic attack" OR "transient ischaemic attack" OR "transient ischaemic attacks" OR "TIA" OR "TIAs" OR "transient brainstem ischemia" OR "transient brainstem ischaemia" OR "transient brain stem ischemia" OR "transient brain stem ischaemia" OR "transient brain ischemia" OR "transient brain ischaemia" OR "transient cerebral ischemia" OR "transient cerebral ischaemia" OR "transient cerebral ischemic" OR "transient cerebral ischaemic" OR ("transient" AND ("brain" OR "cerebral") AND (ischemi* OR ischaem*))) AND ti=("Sex" OR "Gender" OR "Sex" OR "Sexual Characteristics" OR "Sex Characteristic*" OR "Sex Difference*" OR "Gender Difference*" OR "Sex Based" OR "Sex Dimorphism*" OR "Sexual Dimorphism*" OR (("Female" OR "Women" OR "Woman" OR "Female") AND ("Male" OR "Men" OR "Man" OR "Male"))) AND ts=("Pain" OR "pain" OR "facial pain" OR "Confusion" OR "disorientation" OR disorient* OR "confusion" OR confus* OR "Unconsciousness" OR "Unconsciousness" OR "loss of consciousness" OR "Consciousness Disorder" OR "decreased consciousness" OR "Dizziness" OR "Dizziness" OR "lightheadedness" OR "headache" OR headach* OR "Nausea" OR "Nausea" OR "nonfocal weakness" OR "non-focal weakness" OR "nonfocal symptom" OR "non-focal symptom" OR "nonfocal symptoms" OR "non-focal symptoms" OR "non-neurological symptoms" OR "Thorax Pain" OR "chest pain" OR "palpitations" OR "palpitation" OR "Dyspnea" OR "Dyspnea" OR "shortness of breath" OR "hemi-body numbness" OR "hemibody numbness" OR "numbness" OR "Hypesthesia" OR hypesthesi* OR "Diplopia" OR "diplopia" OR "Visual Disorder" OR "Vision Disorders" OR "Vision Disorder" OR "visual disturbances" OR "visual disturbance" OR "vision disturbances" OR "vision disturbance" OR "Blindness" OR "Blindness" OR "Hemianopsia" OR "hemianopsia" OR "Aphasia" OR "aphasia" OR "Dysarthria" OR "dysarthria" OR "discoordination" OR "Ataxia" OR "ataxia" OR "hemiparesis" OR "haemiparesis" OR "Paresis" OR "Paresis" OR "facial weakness" OR "Facial Paralysis" OR "Facial Paralysis" OR "Vertigo" OR "vertigo" OR "Change in

vision" OR "vision change" OR "vision changes" OR "Vision problem" OR "Hemianopia" OR "Blurred vision" OR "Blurry Vision" OR "Decreased Vision" OR "Double Vision" OR "Vision Alteration" OR "Visual Deficit" OR "Visual Deficits" OR "Visual Field Defect" OR "Visual Field Defects" OR "Visual Field Loss" OR "Loss of Vision" OR "vision loss" OR "Monocular Blindness" OR "Consciousness" OR conscious* OR "drowsy" OR drows* OR "visual field disturbances" OR "visual field disturbance" OR "dysphagia" OR "Dysphagia" OR "clinical course")) OR (ti=("Stroke" OR "Stroke" OR "strokes" OR "CVA" OR "CVAs" OR "Cerebrovascular Accident" OR "Cerebrovascular Accidents" OR "Cerebrovascular Insult" OR "Cerebrovascular Insults" OR "CVI" OR "CVIs" OR "Brain Attack" OR "Brain Attacks" OR "Brain Infarct*" OR "Brain Infarction" OR "Cerebrovascular Apoplexy" OR "Apoplexy" OR "Brain Vascular Accident" OR "Brain Vascular Accidents" OR "Cerebrovascular Stroke" OR "Cerebrovascular Strokes" OR "Cerebral Stroke" OR "Cerebral Strokes" OR "Acute Stroke" OR "Acute Strokes" OR "Acute Cerebrovascular Accident" OR "Acute Cerebrovascular Accidents" OR "Cerebrovascular Event" OR "Cerebrovascular Events" OR "Cerebrovascular Attack" OR "Cerebrovascular Attacks" OR "Cerebral infarct*" OR "Brain ischemia" OR "Brain ischemia" OR "Brain ischaemia" OR "Cerebral Ischemia" OR "Cerebral Ischaemia" OR "Cerebral Ischaemia" OR "Brain Hemorrhage" OR "Cerebral Hemorrhag*" OR "Cerebral Haemorrhag*" OR "Intracerebral Hemorrhag*" OR "Brain Hemorrhag*" OR "Brain Haemorrhag*" OR "Intracerebral Haemorrhag*" OR "Neurologic Event" OR "Neurologic Events" OR "Transient Ischemic Attack, Transient" OR "transient ischemic attack" OR "transient ischemic attack" OR "transient ischaemic attack" OR "transient ischaemic attacks" OR "TIA" OR "TIAs" OR "transient brainstem ischemia" OR "transient brainstem ischaemia" OR "transient brain stem ischemia" OR "transient brain stem ischaemia" OR "transient brain ischemia" OR "transient brain ischaemia" OR "transient cerebral ischemia" OR "transient cerebral ischaemia" OR "transient cerebral ischemic" OR "transient cerebral ischaemic" OR ("transient" AND ("brain" OR "cerebral") AND (ischemi* OR ischaem*))) AND ti=("Sex" OR "Gender" OR "Sex" OR "sexes" OR "Sexual Characteristics" OR "Sex Characteristic*" OR "Sex Difference*" OR "Gender Difference*" OR "Sex Based" OR "Sex Dimorphism*" OR "Sexual Dimorphism*" OR ((*"Female" OR "Women" OR "Woman" OR "Female") AND (*"Male" OR "Men" OR "Man" OR "Male"))) AND ts=((differenc* OR disparit* OR sex differenc* OR "sex disparit*" OR "gender differenc*" OR "gender disparit*" OR "differences between men and women") AND ("symptom" OR "symptoms")))) NOT ti=(("Case Report" OR "case report" OR "Review" OR "review") NOT ("Clinical Study" OR "Clinical Trial" OR "trial" OR "RCT"))

IV. The Cochrane Library

(Final search run on 27-05-2020; 335 items)

((("cerebrovascular accident" OR "Stroke" OR "strokes" OR "CVA" OR "CVAs" OR "Cerebrovascular Accident" OR "Cerebrovascular Accidents" OR "Cerebrovascular Insult" OR "Cerebrovascular Insults" OR "CVI" OR "CVIs" OR "Brain Attack" OR "Brain Attacks" OR "Brain Infarct*" OR "Brain Infarction" OR "Cerebrovascular Apoplexy" OR "Apoplexy" OR "Brain Vascular Accident" OR "Brain Vascular Accidents" OR "Cerebrovascular Stroke" OR "Cerebrovascular Strokes" OR "Cerebral Stroke" OR "Cerebral Strokes" OR "Acute Stroke" OR "Acute Strokes" OR "Acute Cerebrovascular Accident" OR "Acute Cerebrovascular Accidents" OR "Cerebrovascular Event" OR "Cerebrovascular Events" OR "Cerebrovascular Attack" OR "Cerebrovascular Attacks" OR "Cerebral infarct*" OR "Brain ischemia" OR "Brain ischemia" OR "Brain ischaemia" OR "Cerebral Ischemia" OR "Cerebral Ischaemia" OR "Brain Hemorrhage" OR "Cerebral Hemorrhag*" OR "Cerebral Haemorrhag*" OR "Intracerebral Hemorrhag*" OR "Brain Hemorrhag*" OR "Brain Haemorrhag*" OR "Intracerebral Haemorrhag*" OR "Neurologic Event" OR "Neurologic Events" OR "Transient Ischemic Attack" OR "transient ischemic attack" OR "transient ischemic attack" OR "transient ischaemic attack" OR "transient ischaemic attacks" OR "TIA" OR "TIAs" OR "transient brainstem ischemia" OR "transient brainstem ischaemia" OR "transient brain stem ischemia" OR "transient brain stem ischaemia" OR "transient brain ischemia" OR "transient brain ischaemia" OR "transient cerebral ischemia" OR "transient cerebral ischaemia" OR "transient cerebral ischemic" OR "transient cerebral ischaemic" OR ("transient" AND ("brain" OR "cerebral") AND (ischemi* OR ischaem*))):ti AND ("Sex" OR "Gender" OR "Sex" OR "Sexual Characteristics" OR "Sex Characteristic*" OR "Sex Difference*" OR "Gender Difference*" OR "Sex Based" OR "Sex Dimorphism*" OR "Sexual Dimorphism*" OR (("Female" OR "Women" OR "Woman" OR "Female") AND ("Male" OR "Men" OR "Man" OR "Male"))):ti AND ("Pain" OR "pain" OR "facial pain" OR "Confusion" OR "disorientation" OR disorient* OR "confusion" OR confus* OR "Unconsciousness" OR "Unconsciousness" OR "loss of consciousness" OR "Consciousness Disorder" OR "decreased consciousness" OR "Dizziness" OR "Dizziness" OR "lightheadedness" OR "headache" OR headach* OR "Nausea" OR "Nausea" OR "nonfocal weakness" OR "non-focal weakness" OR "nonfocal symptom" OR "non-focal symptom" OR "nonfocal symptoms" OR "non-focal symptoms" OR "non-neurological symptoms" OR "Thorax Pain" OR "chest pain" OR "palpitations" OR "palpitation" OR "Dyspnea" OR "Dyspnea" OR "shortness of breath" OR "hemi-body numbness" OR "hemibody numbness" OR "numbness" OR "Hypesthesia" OR hypesthesi* OR "Diplopia" OR "diplopia" OR "Visual Disorder" OR "Vision Disorders" OR "Vision Disorder" OR "visual disturbances" OR "visual disturbance" OR "vision disturbances" OR "vision disturbance" OR "Blindness" OR "Blindness" OR "Hemianopsia" OR "hemianopsia" OR "Aphasia" OR "aphasia" OR "Dysarthria" OR "dysarthria" OR "discoordination" OR "Ataxia" OR "ataxia" OR "hemiparesis" OR "haemiparesis" OR "Paresis" OR "Paresis" OR "facial weakness" OR "Facial Paralysis" OR "Facial Paralysis" OR "Vertigo" OR "vertigo" OR "Change in vision" OR "vision change" OR "vision changes" OR "Vision problem" OR "Hemianopia" OR "Blurred vision" OR "Blurry Vision" OR "Decreased Vision" OR

"Double Vision" OR "Vision Alteration" OR "Visual Deficit" OR "Visual Deficits" OR "Visual Field Defect" OR "Visual Field Defects" OR "Visual Field Loss" OR "Loss of Vision" OR "vision loss" OR "Monocular Blindness" OR "Consciousness" OR conscious* OR "drowsy" OR drows* OR "visual field disturbances" OR "visual field disturbance" OR "dysphagia" OR "Dysphagia" OR "clinical course"):ti,ab,kw) OR (("cerebrovascular accident" OR "Stroke" OR "strokes" OR "CVA" OR "CVAs" OR "Cerebrovascular Accident" OR "Cerebrovascular Accidents" OR "Cerebrovascular Insult" OR "Cerebrovascular Insults" OR "CVI" OR "CVIs" OR "Brain Attack" OR "Brain Attacks" OR "Brain Infarct*" OR "Brain Infarction" OR "Cerebrovascular Apoplexy" OR "Apoplexy" OR "Brain Vascular Accident" OR "Brain Vascular Accidents" OR "Cerebrovascular Stroke" OR "Cerebrovascular Strokes" OR "Cerebral Stroke" OR "Cerebral Strokes" OR "Acute Stroke" OR "Acute Strokes" OR "Acute Cerebrovascular Accident" OR "Acute Cerebrovascular Accidents" OR "Cerebrovascular Event" OR "Cerebrovascular Events" OR "Cerebrovascular Attack" OR "Cerebrovascular Attacks" OR "Cerebral infarct*" OR "Brain ischemia" OR "Brain ischemia" OR "Brain ischaemia" OR "Cerebral Ischemia" OR "Cerebral Ischaemia" OR "Brain Hemorrhage" OR "Cerebral Hemorrhag*" OR "Cerebral Haemorrhag*" OR "Intracerebral Hemorrhag*" OR "Brain Hemorrhag*" OR "Brain Haemorrhag*" OR "Intracerebral Haemorrhag*" OR "Neurologic Event" OR "Neurologic Events" OR "Transient Ischemic Attack" OR "transient ischemic attack" OR "transient ischemic attack" OR "transient ischaemic attack" OR "transient ischaemic attacks" OR "TIA" OR "TIAs" OR "transient brainstem ischemia" OR "transient brainstem ischaemia" OR "transient brain stem ischemia" OR "transient brain stem ischaemia" OR "transient brain ischemia" OR "transient brain ischaemia" OR "transient cerebral ischemia" OR "transient cerebral ischaemia" OR "transient cerebral ischemic" OR "transient cerebral ischaemic" OR ("transient" AND ("brain" OR "cerebral") AND (ischemi* OR ischaem*))):ti AND ("Sex" OR "Gender" OR "Sex" OR "Sexual Characteristics" OR "Sex Characteristic*" OR "Sex Difference*" OR "Gender Difference*" OR "Sex Based" OR "Sex Dimorphism*" OR "Sexual Dimorphism*" OR (("Female" OR "Women" OR "Woman" OR "Female") AND ("Male" OR "Men" OR "Man" OR "Male"))):ti,ab,kw AND ("Pain" OR "pain" OR "facial pain" OR "Confusion" OR "disorientation" OR disorient* OR "confusion" OR confus* OR "Unconsciousness" OR "Unconsciousness" OR "loss of consciousness" OR "Consciousness Disorder" OR "decreased consciousness" OR "Dizziness" OR "Dizziness" OR "lightheadedness" OR "headache" OR headach* OR "Nausea" OR "Nausea" OR "nonfocal weakness" OR "non-focal weakness" OR "nonfocal symptom" OR "non-focal symptom" OR "nonfocal symptoms" OR "non-focal symptoms" OR "non-neurological symptoms" OR "Thorax Pain" OR "chest pain" OR "palpitations" OR "palpitation" OR "Dyspnea" OR "Dyspnea" OR "shortness of breath" OR "hemi-body numbness" OR "hemibody numbness" OR "numbness" OR "Hypesthesia" OR hypesthesi* OR "Diplopia" OR "diplopia" OR "Visual Disorder" OR "Vision Disorders" OR "Vision Disorder" OR "visual disturbances" OR "visual disturbance" OR "vision disturbances" OR "vision disturbance" OR "Blindness" OR "Blindness" OR "Hemianopsia" OR "hemianopsia" OR "Aphasia" OR "aphasia" OR "Dysarthria" OR "dysarthria" OR "discoordination" OR "Ataxia" OR "ataxia" OR "hemiparesis" OR "haemiparesis" OR "Paresis" OR "Paresis" OR "facial weakness" OR "Facial Paralysis" OR "Facial Paralysis" OR "Vertigo" OR "vertigo" OR "Change in

vision" OR "vision change" OR "vision changes" OR "Vision problem" OR "Hemianopia" OR "Blurred vision" OR "Blurry Vision" OR "Decreased Vision" OR "Double Vision" OR "Vision Alteration" OR "Visual Deficit" OR "Visual Deficits" OR "Visual Field Defect" OR "Visual Field Defects" OR "Visual Field Loss" OR "Loss of Vision" OR "vision loss" OR "Monocular Blindness" OR "Consciousness" OR conscious* OR "drowsy" OR drows* OR "visual field disturbances" OR "visual field disturbance" OR "dysphagia" OR "Dysphagia" OR "clinical course"):ti) OR (("Stroke" OR "Stroke" OR "strokes" OR "CVA" OR "CVAs" OR "Cerebrovascular Accident" OR "Cerebrovascular Accidents" OR "Cerebrovascular Insult" OR "Cerebrovascular Insults" OR "CVI" OR "CVIs" OR "Brain Attack" OR "Brain Attacks" OR "Brain Infarct*" OR "Brain Infarction" OR "Cerebrovascular Apoplexy" OR "Apoplexy" OR "Brain Vascular Accident" OR "Brain Vascular Accidents" OR "Cerebrovascular Stroke" OR "Cerebrovascular Strokes" OR "Cerebral Stroke" OR "Cerebral Strokes" OR "Acute Stroke" OR "Acute Strokes" OR "Acute Cerebrovascular Accident" OR "Acute Cerebrovascular Accidents" OR "Cerebrovascular Event" OR "Cerebrovascular Events" OR "Cerebrovascular Attack" OR "Cerebrovascular Attacks" OR "Cerebral infarct*" OR "Brain ischemia" OR "Brain ischemia" OR "Brain ischaemia" OR "Cerebral Ischemia" OR "Cerebral Ischaemia" OR "Cerebral Ischaemia" OR "Brain Hemorrhage" OR "Cerebral Hemorrhag*" OR "Cerebral Haemorrhag*" OR "Intracerebral Hemorrhag*" OR "Brain Hemorrhag*" OR "Brain Haemorrhag*" OR "Intracerebral Haemorrhag*" OR "Neurologic Event" OR "Neurologic Events" OR "Transient Ischemic Attack, Transient" OR "transient ischemic attack" OR "transient ischemic attack" OR "transient ischaemic attack" OR "transient ischaemic attacks" OR "TIA" OR "TIAs" OR "transient brainstem ischemia" OR "transient brainstem ischaemia" OR "transient brain stem ischemia" OR "transient brain stem ischaemia" OR "transient brain ischemia" OR "transient brain ischaemia" OR "transient cerebral ischemia" OR "transient cerebral ischaemia" OR "transient cerebral ischemic" OR "transient cerebral ischaemic" OR ("transient" AND ("brain" OR "cerebral") AND (ischemi* OR ischaem*))):ti AND ("Sex" OR "Gender" OR "Sex" OR "sexes" OR "Sexual Characteristics" OR "Sex Characteristic*" OR "Sex Difference*" OR "Gender Difference*" OR "Sex Based" OR "Sex Dimorphism*" OR "Sexual Dimorphism*" OR ((*"Female" OR "Women" OR "Woman" OR "Female") AND (*"Male" OR "Men" OR "Man" OR "Male"))):ti AND ((differenc* OR disparit* OR sex differenc* OR "sex disparit*" OR "gender differenc*" OR "gender disparit*" OR "differences between men and women") AND ("symptom" OR "symptoms")):ti,ab,kw))

V. Emcare

(Final search run on 27-05-2020; 327 items)

(((exp *"cerebrovascular accident"/ OR "Stroke".ti OR "strokes".ti OR "CVA".ti OR "CVAs".ti OR "Cerebrovascular Accident".ti OR "Cerebrovascular Accidents".ti OR "Cerebrovascular Insult".ti OR "Cerebrovascular Insults".ti OR "CVI".ti OR "CVIs".ti OR "Brain Attack".ti OR "Brain Attacks".ti OR "Brain Infarct*".ti OR exp *"Brain Infarction"/ OR "Cerebrovascular Apoplexy".ti OR "Apoplexy".ti OR "Brain Vascular Accident".ti OR "Brain Vascular Accidents".ti OR "Cerebrovascular Stroke".ti OR "Cerebrovascular Strokes".ti OR "Cerebral Stroke".ti OR "Cerebral Strokes".ti OR "Acute Stroke".ti OR "Acute Strokes".ti OR "Acute Cerebrovascular Accident".ti OR "Acute Cerebrovascular Accidents".ti OR "Cerebrovascular Event".ti OR "Cerebrovascular Events".ti OR "Cerebrovascular Attack".ti OR "Cerebrovascular Attacks".ti OR "Cerebral infarct*".ti OR exp *"Brain ischemia"/ OR "Brain ischemia".ti OR "Brain ischaemia".ti OR "Cerebral Ischemia".ti OR "Cerebral Ischaemia".ti OR exp *"Brain Hemorrhage"/ OR "Cerebral Hemorrhag*".ti OR "Cerebral Haemorrhag*".ti OR "Intracerebral Hemorrhag*".ti OR "Brain Hemorrhag*".ti OR "Brain Haemorrhag*".ti OR "Intracerebral Haemorrhag*".ti OR "Neurologic Event".ti OR "Neurologic Events".ti OR "Transient Ischemic Attack"/ OR "transient ischemic attack".ti OR "transient ischemic attack".ti OR "transient ischaemic attack".ti OR "transient ischaemic attacks".ti OR "TIA".ti OR "TIAs".ti OR "transient brainstem ischemia".ti OR "transient brainstem ischaemia".ti OR "transient brain stem ischemia".ti OR "transient brain stem ischaemia".ti OR "transient brain ischemia".ti OR "transient brain ischaemia".ti OR "transient cerebral ischemia".ti OR "transient cerebral ischaemia".ti OR "transient cerebral ischemic".ti OR "transient cerebral ischaemic".ti OR ("transient".ti ADJ3 ("brain".ti OR "cerebral".ti) ADJ3 (ischemi*.ti OR ischaem*.ti))) AND (*"Sex"/ OR "Gender".ti,ab OR "Sex".ti,ab OR exp *"Sexual Characteristics"/ OR "Sex Characteristic*".ti,ab OR "Sex Difference*".ti,ab OR "Gender Difference*".ti,ab OR "Sex Based".ti,ab OR "Sex Dimorphism*".ti,ab OR "Sexual Dimorphism*".ti,ab OR (("Women".ti,ab OR "Woman".ti,ab OR "Female".ti,ab) AND ("Men".ti,ab OR "Man".ti,ab OR "Male".ti,ab))) AND (exp *"Pain"/ OR "pain".ti OR "facial pain".ti OR "Confusion"/ OR "disorientation".ti OR disorient*.ti OR "confusion".ti OR confus*.ti OR exp *"Unconsciousness"/ OR "Unconsciousness".ti OR "loss of consciousness".ti OR exp *"Consciousness Disorder"/ OR "decreased consciousness".ti OR "Dizziness"/ OR "Dizziness".ti OR "lightheadedness".ti OR exp *"headache"/ OR "headache".ti OR headach*.ti OR exp *"Nausea"/ OR "Nausea".ti OR "nonfocal weakness".ti OR "non-focal weakness".ti OR "nonfocal symptom".ti OR "non-focal symptom".ti OR "nonfocal symptoms".ti OR "non-focal symptoms".ti OR "non-neurological symptoms".ti OR "Thorax Pain"/ OR "chest pain".ti OR "palpitations".ti OR "palpitation".ti OR exp *"Dyspnea"/ OR "Dyspnea".ti OR "shortness of breath" .ti OR "hemi-body numbness".ti OR "hemibody numbness".ti OR "numbness".ti OR exp *"Hypesthesia"/ OR hypesthesi*.ti OR exp *"Diplopia"/ OR "diplopia".ti OR "Visual Disorder"/ OR "Vision Disorders".ti OR "Vision Disorder".ti OR "visual disturbances".ti OR "visual disturbance".ti OR "vision disturbances".ti OR "vision disturbance".ti OR exp *"Blindness"/ OR "Blindness".ti OR exp *"Hemianopsia"/ OR "hemianopsia".ti OR exp *"Aphasia"/ OR "aphasia".ti OR exp *"Dysarthria"/ OR "dysarthria".ti OR

"discoordination".ti OR exp *"Ataxia"/ OR "ataxia".ti OR "hemiparesis".ti OR "haemiparesis".ti OR exp *"Paresis"/ OR "Paresis".ti OR "facial weakness".ti OR exp *"Facial Paralysis"/ OR "Facial Paralysis".ti OR exp *"Vertigo"/ OR "vertigo".ti OR "Change in vision".ti OR "vision change".ti OR "vision changes".ti OR "Vision problem".ti OR "Hemianopia".ti OR "Blurred vision".ti OR "Blurry Vision".ti OR "Decreased Vision".ti OR "Double Vision".ti OR "Vision Alteration".ti OR "Visual Deficit".ti OR "Visual Deficits".ti OR "Visual Field Defect".ti OR "Visual Field Defects".ti OR "Visual Field Loss".ti OR "Loss of Vision".ti OR "vision loss".ti OR "Monocular Blindness".ti OR exp *"Consciousness"/ OR conscious*.ti OR "drowsy".ti OR drows*.ti OR "visual field disturbances".ti OR "visual field disturbance".ti OR "dysphagia".ti OR exp *"Dysphagia"/ OR "clinical course".ti)) OR ((exp *"cerebrovascular accident"/ OR "Stroke".ti OR "strokes".ti OR "CVA".ti OR "CVAs".ti OR "Cerebrovascular Accident".ti OR "Cerebrovascular Accidents".ti OR "Cerebrovascular Insult".ti OR "Cerebrovascular Insults".ti OR "CVI".ti OR "CVIs".ti OR "Brain Attack".ti OR "Brain Attacks".ti OR "Brain Infarct*".ti OR exp *"Brain Infarction"/ OR "Cerebrovascular Apoplexy".ti OR "Apoplexy".ti OR "Brain Vascular Accident".ti OR "Brain Vascular Accidents".ti OR "Cerebrovascular Stroke".ti OR "Cerebrovascular Strokes".ti OR "Cerebral Stroke".ti OR "Cerebral Strokes".ti OR "Acute Stroke".ti OR "Acute Strokes".ti OR "Acute Cerebrovascular Accident".ti OR "Acute Cerebrovascular Accidents".ti OR "Cerebrovascular Event".ti OR "Cerebrovascular Events".ti OR "Cerebrovascular Attack".ti OR "Cerebrovascular Attacks".ti OR "Cerebral infarct*".ti OR exp *"Brain ischemia"/ OR "Brain ischemia".ti OR "Brain ischaemia".ti OR "Cerebral Ischemia".ti OR "Cerebral Ischaemia".ti OR exp *"Brain Hemorrhage"/ OR "Cerebral Hemorrhag*".ti OR "Cerebral Haemorrhag*".ti OR "Intracerebral Hemorrhag*".ti OR "Brain Hemorrhag*".ti OR "Brain Haemorrhag*".ti OR "Intracerebral Haemorrhag*".ti OR "Neurologic Event".ti OR "Neurologic Events".ti OR "Transient Ischemic Attack"/ OR "transient ischemic attack".ti OR "transient ischemic attack".ti OR "transient ischaemic attack".ti OR "transient ischaemic attacks".ti OR "TIA".ti OR "TIAs".ti OR "transient brainstem ischemia".ti OR "transient brainstem ischaemia".ti OR "transient brain stem ischemia".ti OR "transient brain stem ischaemia".ti OR "transient brain ischemia".ti OR "transient brain ischaemia".ti OR "transient cerebral ischemia".ti OR "transient cerebral ischaemia".ti OR "transient cerebral ischemic".ti OR "transient cerebral ischaemic".ti OR ("transient".ti ADJ3 ("brain".ti OR "cerebral".ti) ADJ3 (ischemi*.ti OR ischaem*.ti))) AND (*"Sex"/ OR "Gender".ti OR "Sex".ti OR exp *"Sexual Characteristics"/ OR "Sex Characteristic*".ti OR "Sex Difference*".ti OR "Gender Difference*".ti OR "Sex Based".ti OR "Sex Dimorphism*".ti OR "Sexual Dimorphism*".ti OR (("Women".ti OR "Woman".ti OR "Female".ti) AND ("Men".ti OR "Man".ti OR "Male".ti))) AND (exp *"Pain"/ OR "pain".ti,ab OR "facial pain".ti,ab OR "Confusion"/ OR "disorientation".ti,ab OR disorient*.ti,ab OR "confusion".ti,ab OR confus*.ti,ab OR exp *"Unconsciousness"/ OR "Unconsciousness".ti,ab OR "loss of consciousness".ti,ab OR exp *"Consciousness Disorder"/ OR "decreased consciousness".ti,ab OR "Dizziness"/ OR "Dizziness".ti,ab OR "lightheadedness".ti,ab OR exp *"headache"/ OR "headache".ti,ab OR headach*.ti,ab OR exp *"Nausea"/ OR "Nausea".ti,ab OR "nonfocal weakness".ti,ab OR "non-focal weakness".ti,ab OR "nonfocal symptom".ti,ab OR "non-focal symptom".ti,ab OR "nonfocal symptoms".ti,ab OR "non-focal symptoms".ti,ab OR "non-neurological

symptoms".ti,ab OR "Thorax Pain"/ OR "chest pain".ti,ab OR "palpitations".ti,ab OR "palpitation".ti,ab OR exp *"Dyspnea"/ OR "Dyspnea".ti,ab OR "shortness of breath" .ti,ab OR "hemi-body numbness".ti,ab OR "hemibody numbness".ti,ab OR "numbness".ti,ab OR exp *"Hypesthesia"/ OR hypesthesi*.ti,ab OR exp *"Diplopia"/ OR "diplopia".ti,ab OR "Visual Disorder"/ OR "Vision Disorders".ti,ab OR "Vision Disorder".ti,ab OR "visual disturbances".ti,ab OR "visual disturbance".ti,ab OR "vision disturbances".ti,ab OR "vision disturbance".ti,ab OR exp *"Blindness"/ OR "Blindness".ti,ab OR exp *"Hemianopsia"/ OR "hemianopsia".ti,ab OR exp *"Aphasia"/ OR "aphasia".ti,ab OR exp *"Dysarthria"/ OR "dysarthria".ti,ab OR "discoordination".ti,ab OR exp *"Ataxia"/ OR "ataxia".ti,ab OR "hemiparesis".ti,ab OR "haemiparesis".ti,ab OR exp *"Paresis"/ OR "Paresis".ti,ab OR "facial weakness".ti,ab OR exp *"Facial Paralysis"/ OR "Facial Paralysis".ti,ab OR exp *"Vertigo"/ OR "vertigo".ti,ab OR "Change in vision".ti,ab OR "vision change".ti,ab OR "vision changes".ti,ab OR "Vision problem".ti,ab OR "Hemianopia".ti,ab OR "Blurred vision".ti,ab OR "Blurry Vision".ti,ab OR "Decreased Vision".ti,ab OR "Double Vision".ti,ab OR "Vision Alteration".ti,ab OR "Visual Deficit".ti,ab OR "Visual Deficits".ti,ab OR "Visual Field Defect".ti,ab OR "Visual Field Defects".ti,ab OR "Visual Field Loss".ti,ab OR "Loss of Vision".ti,ab OR "vision loss".ti,ab OR "Monocular Blindness".ti,ab OR exp *"Consciousness"/ OR conscious*.ti,ab OR "drowsy".ti,ab OR drows*.ti,ab OR "visual field disturbances".ti,ab OR "visual field disturbance".ti,ab OR "dysphagia".ti,ab OR exp *"Dysphagia"/ OR "clinical course".ti,ab)) OR ((exp *"Stroke"/ OR "Stroke".ti OR "strokes".ti OR "CVA".ti OR "CVAs".ti OR "Cerebrovascular Accident".ti OR "Cerebrovascular Accidents".ti OR "Cerebrovascular Insult".ti OR "Cerebrovascular Insults".ti OR "CVI".ti OR "CVIs".ti OR "Brain Attack".ti OR "Brain Attacks".ti OR "Brain Infarct*".ti OR exp *"Brain Infarction"/ OR "Cerebrovascular Apoplexy".ti OR "Apoplexy".ti OR "Brain Vascular Accident".ti OR "Brain Vascular Accidents".ti OR "Cerebrovascular Stroke".ti OR "Cerebrovascular Strokes".ti OR "Cerebral Stroke".ti OR "Cerebral Strokes".ti OR "Acute Stroke".ti OR "Acute Strokes".ti OR "Acute Cerebrovascular Accident".ti OR "Acute Cerebrovascular Accidents".ti OR "Cerebrovascular Event".ti OR "Cerebrovascular Events".ti OR "Cerebrovascular Attack".ti OR "Cerebrovascular Attacks".ti OR "Cerebral infarct*".ti OR exp *"Brain ischemia"/ OR "Brain ischemia".ti OR "Brain ischaemia".ti OR "Cerebral Ischemia".ti OR "Cerebral Ischaemia".ti OR exp *"Brain Hemorrhage"/ OR "Cerebral Hemorrhag*".ti OR "Cerebral Haemorrhag*".ti OR "Intracerebral Hemorrhag*".ti OR "Brain Hemorrhag*".ti OR "Brain Haemorrhag*".ti OR "Intracerebral Haemorrhag*".ti OR "Neurologic Event".ti OR "Neurologic Events".ti OR "Transient Ischemic Attack, Transient"/ OR "transient ischemic attack".ti OR "transient ischemic attack".ti OR "transient ischaemic attack".ti OR "transient ischaemic attacks".ti OR "TIA".ti OR "TIAs".ti OR "transient brainstem ischemia".ti OR "transient brainstem ischaemia".ti OR "transient brain stem ischemia".ti OR "transient brain stem ischaemia".ti OR "transient brain ischemia".ti OR "transient brain ischaemia".ti OR "transient cerebral ischemia".ti OR "transient cerebral ischaemia".ti OR "transient cerebral ischemic".ti OR "transient cerebral ischaemic".ti OR ("transient".ti ADJ3 ("brain".ti OR "cerebral".ti) ADJ3 (ischemi*.ti OR ischaem*.ti))) AND (*"Sex"/ OR "Gender".ti OR "Sex".ti OR "sexes".ti OR exp *"Sexual Characteristics"/ OR "Sex Characteristic*".ti OR "Sex Difference*".ti OR "Gender Difference*".ti OR "Sex

Based".ti OR "Sex Dimorphism*".ti OR "Sexual Dimorphism*".ti OR ((*"Female"/ OR "Women".ti OR "Woman".ti OR "Female".ti) AND (*"Male"/ OR "Men".ti OR "Man".ti OR "Male".ti))) AND (differenc*.ti OR disparit*.ti OR sex differenc*.ti,ab OR sex disparit*.ti,ab OR gender differenc*.ti,ab OR gender disparit*.ti,ab OR "differences between men and women".ti,ab) AND ("symptom".ti,ab OR "symptoms".ti,ab))) NOT (("Case Report"/ OR "case report".ti OR exp "Review"/ OR "review".ti) NOT ("Clinical Study"/ OR exp "Clinical Trial"/ OR "trial".ti OR "RCT".ti))

Supplement Method. Protocol - risk of bias assessment according to the Newcastle-Ottawa Scale (customized version)

1) Validation of diagnosis.
Low risk of bias for ischemic and hemorrhagic stroke diagnosis was defined as a confirmed stroke on neuroimaging (MRI or CT). Low risk of bias for TIA patients was defined as evaluation of TIA in a TIA clinic by an emergency medicine doctor or a neurologist (in training). High risk of bias was defined as stroke or TIA assessment not conducted as specified by the aforementioned methods in all patients (such as use of the International Classification of Diseases codes [ICD]), unclear assessment or assessment by emergency medical dispatchers.

2) Assessment of symptoms.
Low risk of bias was defined as description of neurological examination and questioning through structured interviews using a standardized questionnaire during first neurological examination at the emergency department or during hospital admission. The medical history and the neurological examination needed to be conducted by an emergency medicine doctor or a neurologist (in training). Whenever a speech therapist examined language disorders such as aphasia during hospital admission, this was also defined as low risk of bias.
High risk of bias was classified as retrospective symptom assessment instead of assessments of symptoms during admission for stroke, or as the collection of data on symptom presentation directly from electronic health records (EHR). Collection of data on stroke symptoms did not have to be for the sole purpose of studying sex differences, as sex is in principle accurately recorded in EHR regardless of research question.

3) Adjustment for confounding.
Adjustment for age and/or stroke type was allocated as low risk of bias. High risk of bias was classified as unadjusted comparisons between sexes.

4) Generalizability.
Stroke specific inclusion criteria were examined to assess selection bias. Studies including (1) all subtypes of stroke and (2) a patient population that is a random subsample of an average stroke population (i.e. no restrictions for patients with certain stroke etiologies to be included), were categorized as low risk of bias. Studies with populations that were restricted by (1) and/or (2) were specified as high risk of bias. Moreover, if no clear distinction was made between SAH and ICH, this was defined as high risk of bias as well.
For each element of the risk of bias analysis, studies were defined as low or high risk. Possible risk of bias was reported when data on this element in the risk of bias assessment was missing or unclear.

Supplement Table I. Characteristics of included studies

Nr.	1. Author, year	2. Study center, country	3. Study period	3. N	4. Women, n (%)	5. Men, n (%)	6. Mean age women/men, y	7. Stroke subtype	8. Study design
1	Abadie, 2014	Dijon Stroke Registry, Dijon University Hospital and three private hospitals of the city, Dijon, France	1 January 2006-31 December 2011	1,411	777 (55%)	634 (45%)	NA	Ischemic stroke, ICH, and TIA. Exclusion: SAH (analyses for ischemic and hemorrhagic stroke performed separately)	Population-based stroke registry, prospective, multicenter
2	Acciarresi, 2014	Perugia Stroke Registry, Stroke Unit at the University of Perugia, Italy	June 1, 2005-May 2012	1,883	1,072 (57%)	811 (43%)	75/70	Ischemic or hemorrhagic stroke or TIA	Registry cohort, prospective, single-center
3	Ahmadi Aghangar, 2015	3 hospitals in Babol, Iran	April 1 2011-31 June 2012	263	124 (47%)	139 (53%)	NA	Ischemic stroke	Observational study, prospective, multicenter
4	Alves, 2012	19 hospitals in Fortaleza, Brazil	June 2009-October 2010	364	191 (52%)	173 (48%)	66/59	ICH	Hospital-based study, prospective, multicenter
5	Arboix, 2001	Barcelona Stroke Registry, Sagrat Cor-L'Alianza Hospital, Barcelona, Spain	January 1986-December 1995	2	1,033 (52%)	967 (48%)	75/69	TIA, atherothrombotic infarct, lacunar stroke, cardioembolic infarct, parenchymal hemorrhage, SAH, spontaneous subdural hematoma, and	Hospital-based stroke registry, prospective, single-center

Nr.	**1. Author, year**	**2. Study center, country**	**3. Study period**	**3. N**	**4. Women, n (%)**	**5. Men, n (%)**	**6. Mean age women/men, y**	**7. Stroke subtype**	**8. Study design**
								epidural hematoma	
6	Aziz, 2016	National Neurology Registry (NNEUR), 13 hospitals, Malaysia	July 2009-June 2015	4,762	2,624 (55%)	2,138 (45%)	64/62	Ischemic stroke	Registry cohort, prospective, multicenter
7	Barrett, 2007	the Ischemic Stroke Genetics Study, 5 medical centers, US	NA	505	276 (55%)	229 (45%)	NA	Ischemic stroke	Genetic association study (case-control design), prospective, multicenter
8	Becker, 1986	The Community Hospital-based Stroke Programs (CHSPs), 58 hospitals, three community sites, US	1979-1980	3,334	1,555 (47%)	1,779 (53%)	71/68	Infarct and hemorrhage	Hospital-based stroke registry, prospective, multicenter
9	Bersano, 2009	PROSIT study, seven Italian regions, Italy	2001 (one-year period)	8,848	4,192 (47%)	4,656 (53%)	NA	Ischemic or hemorrhagic stroke	Observational follow-up, prospective, multicenter
10	Brust, 1976	Harlem Regional Stroke Program, Harlem Hospital, New York, US	1971-1973	850	472 (56%)	378 (44%)	NA	NA	Stroke registry, prospective, single-center
11	Chen, 2013	Taiwan Stroke Registry, 39 academic and community hospitals diffusely covering the entire country	August 1, 2006-unclear	11,523	4,503 (39%)	7,020 (61%)	NA	Ischemic stroke	Stroke registry, prospective, multicenter
12	Di Carlo, 2003	European Concerted Action, 7 countries and 22 hospitals	September 1993-September 1994	4,499	2,239 (50%)	2,260 (50%)	75/69	Cerebral infarction, cerebral hemorrhage, SAH, or unclassifiable stroke	Hospital-based stroke register, prospective, multicenter & multinational

Nr.	**1. Author, year**	**2. Study center, country**	**3. Study period**	**3. N**	**4. Women, n (%)**	**5. Men, n (%)**	**6. Mean age women/men, y**	**7. Stroke subtype**	**8. Study design**
13	Elhfnawy, 2020	University Hospital of Würzburg, Würzburg, Germany	February-October 2018	59	21 (36%)	38 (64%)	NA	Brainstem or cerebellar infarctions	Cohort study, prospective, single-center,
14	Engeltelter, 2006	Local university hospital, Basle City, Switzerland	June 1, 2002-May 31, 2003	269	114 (42%)	155 (58%)	NA	Ischemic stroke	Population-based study, prospective, single-center
15	Ferro, 1995	The Hospital Santa Maria Stroke Data Base, Hospital de Santa Maria, Lisbon, Portugal	May 1985-March 1994	205	82 (40%)	123 (60%)	NA	TIA	Hospital-based stroke database, prospective, single-center
16	Ferro, 1997	The Hospital Santa Maria Stroke Data Base, Hospital de Santa Maria, Lisbon, Portugal	1985-1996	423	295 (70%)	128 (30%)	NA	Ischemic stroke	Hospital-based stroke database, prospective, single-center
17	Foerch, 2007	>100 hospitals, data was obtained from a large stroke registry in Germany, provided by the Arbeitsgruppe Schlaganfall Hessen	January 1, 1999-December 31, 2005	53,414	27,095 (51%)	26,319 (49%)	NA	Cerebral infarction or ICH(no TIA)	Hospital-based stroke registry, retrospective, multicenter
18	Gall, 2010	North East Melbourne Stroke Incidence Study (NEMESIS), multiple hospitals, Melbourne, Australia	May 1, 1996-April 30, 1999	1,316	585 (44%)	731 (56%)	76/72	Ischemic stroke, ICH, and SAH	Stroke incidence study, prospective, multicenter
19	Gargano, 2009	Michigan Acute Stroke Care Overview & Treatment Surveillance System, 15 hospitals, US	May-November 2002	1,922	881 (46%)	1,041 (54%)	72/68	Acute stroke or TIA	Statewide stroke registry, prospective, multicenter

Nr.	1. Author, year	2. Study center, country	3. Study period	3. N	4. Women, n (%)	5. Men, n (%)	6. Mean age women/men, y	7. Stroke subtype	8. Study design
20	Gialanella, 2011	One rehabilitation hospital in Brescia, Italy	2001–2007	262	135 (52%)	127 (48%)	NA	Infarct and hemorrhage	Hospital-based stroke registry, prospective, single-center
21	Glader, 2003	Riks-Stroke Registry, 75 hospitals in Sweden	2001 (one-year period)	19,547	9,666 (49%)	9881 (51%)	78/73	All stroke events except TIA and SAH	National stroke registry, prospective, multicenter
22	Godefroy, 2002	Lille acute stroke unit, Lille, France	May 1994-August 1997	308	143 (46%)	165 (54%)	NA	Ischemic and hemorrhagic stroke	Observational study, prospective, single-center
23	Grindal, 1974	One academic hospital, Wake Forest Baptist Medical Center, Winston-Salem, North Carolina, US	1964-1973	160	53 (33%)	107 (67%)	NA	TIA	Cohort study, retrospective, single-center
24	Hier, 1994	The Stroke Data Bank, 4 teaching hospitals, US	July 1983-June 1986	1,805	842 (47%)	963 (53%)	69/65	ICH, ischemic stroke, SAH, or stroke due to other etiology	Hospital-based stroke registry, retrospective, multicenter
25	Inatomi, 2008	Kumamoto Hospital, Kumamoto, Japan	April 1, 2004-March 31, 2006	855	324 (38%)	531 (62%)	NA	Ischemic stroke	Cohort study, retrospective, single-center
26	Jerath, 2011	Rochester Epidemiology Project Medical Record Linkage System, several medical centers and hospitals, Rochester, Minnesota, USA	1985-1989	449	181 (40%)	268 (60%)	79/70	Ischemic stroke	Registry cohort, retrospective, multicenter
27	Kadojić, 2012	University Department of Neurology in Osijek, Croatia	2010 (one-year period)	177	94 (53%)	83 (47%)	NA	Ischemic (large vessel, small vessel,	Cohort study, prospective, single-center

Nr.	1. Author, year	2. Study center, country	3. Study period	3. N	4. Women, n (%)	5. Men, n (%)	6. Mean age women/men, y	7. Stroke subtype	8. Study design
								cardioembolic stroke, other determined stroke, or undetermined stroke)	
28	Kapral, 2005	Registry of the Canadian Stroke Network, 21 urban tertiary care centers and 4 large community hospitals, Canada	Phase 1 (July 2001-February 2002)Phase 2 (June-December 2002)	3,323	1,796 (54%)	1,527 (46%)	73/69 (median)	TIA, ischemic stroke, ICH, SAH, or undefined stroke	Stroke registry, prospective, multicenter
29	Kes, 2016	Dubrava University Hospital, Zagreb, Croatia	2004 (one-year period)	396	186 (47%)	210 (53%)	77/73	Ischemic, hemorrhagic, intraparenchymal hemorrhage, or SAH	Cross-sectional, prospective, single-center
30	Khan, 2018	Hamad Medical Corporation (HMC), Doha, Qatar	September 15, 2004 - September 15, 2005	270	72 (27%)	198 (73%)	61/56	Ischemic, SAH, and hemorrhagic stroke (TIA, subdural, and extradural hematomas, excluded)	Hospital based study, prospective, multicenter
31	Kumral, 1995	Lausanne Stroke Registry, Centre Hospitalier Universitaire Vaudois, public hospital in the Lausanne area, Switzerland	1982-unclear	2,506	944 (38%)	1562 (62%)	NA	Ischemic stroke, hemorrhagic stroke, TIA, and cerebral venous thrombosis	Stroke registry, prospective, single-center

Nr.	1. Author, year	2. Study center, country	3. Study period	3. N	4. Women, n (%)	5. Men, n (%)	6. Mean age women/men, y	7. Stroke subtype	8. Study design
32	Labiche, 2002	The TLL Temple Foundation Stroke Project, 10 community hospitals in nonurban East Texas, US	February 1998-March 2000	1,124	467 (42%)	657 (58%)	74/70	TIA, ischemic stroke, ICH, or SAH	Observational study, prospective, multicenter
33	Lai, 2005	Kansas City Stroke Study, 12 hospitals, US	August 1995-September 1998	459	214 (47%)	245 (53%)	71/69	Cerebral infarction or ICH	Cohort study, prospective, multicenter
34	Laska, 2001	Danderyd Hospital, Danderyd, Sweden	1 June 1993-30 September 1994	106	57 (54%)	49 (46%)	NA	All stroke	Cohort study, prospective, single-center
35	Leira, 2002	15 hospitals in Spain	October 1992-December 1996	241	76 (32%)	165 (68%)	NA	Ischemic stroke	Cohort study, prospective, multicenter
36	Leira, 2005	15 hospitals in Spain	May 1999-April 2001	189	81 (43%)	108 (57%)	NA	Cerebral hemorrhage	Cohort study, prospective, multicenter
37	Li, 2016	Ontario Stroke Registry, 11 stroke centers, Ontario, Canada	July 1, 2003-March 31, 2008	5,991	3,079 (51%)	2,912 (49%)	NA	TIA	Cohort study, prospective, multicenter
38	Lisabeth, 2009	University of Michigan Hospital (academic teaching hospital), Ann Harbor, US	January 2005-December 2007	461	237 (51%)	224 (49%)	68/66 (median)	Ischemic stroke or TIA	Stroke incidence study, prospective, single-center
39	Maino, 2013	The LiLAC (Life Long After Cerebral ischaemia) cohort study was based on the Dutch TIA Trial	February 1986-March 1989	2,473	865 (35%)	1,608 (65%)	NA	TIA or minor ischemic stroke of non-cardioembolic origin included	Cohort study, prospective, multicenter

Nr.	1. Author, year	2. Study center, country	3. Study period	3. N	4. Women, n (%)	5. Men, n (%)	6. Mean age women/men, y	7. Stroke subtype	8. Study design
		(DTT). 24 hospitals in The Netherlands.							
40	Medlin, 2020	Acute STroke Registry and Analysis of Lausanne (ASTRAL), Lausanne University Hospital, Lausanne, Switzerland	March 2003-April 2016	3993	1757 (44%)	2236 (56%)	77/70 (median)	Ischemic stroke	Stroke registry, prospective, single-center
41	Melo, 1996	The Hospital Santa Maria Stroke Data Base, Hospital de Santa Maria, Lisbon, Portugal	March 1993-April 1994	289	119 (41%)	170 (59%)	NA	ICH	Cohort study, prospective, single-center
42	Mitsias, 2006	Henry Ford Hospital Stroke Data Bank, Henry Ford Hospital, Detroit, US	1987-1993	375	164 (44%)	211 (56%)	NA	Ischemic stroke	Hospital-based stroke database, prospective, single-center
43	Mochari-Greenberger, 2015	Get With The Guidelines–Stroke participating hospitals, 1613 hospitals, US	October 2011-March 2014	398,798	197,804 (50%)	200,994 (50%)	NA	Ischemic, SAH, ICH, stroke not otherwise specified	Hospital-based stroke registry, prospective, multicenter
44	Niewada, 2005	International Stroke Trial, 467 hospitals in 36 countries	1992-1996	17,37	9,367 (54%)	8,003 (46%)	74/69	Ischemic stroke	RCT, prospective, multicenter & multinational
45	Park, 2013	23 tertiary and teaching hospital EDs, Korea	January 1-December 31, 2008	6,635	3,805 (57%)	2,830 (43%)	70/64	Ischemic stroke	Observational, prospective, multicenter
46	Pedersen, 1996	The Copenhagen Stroke Study, Bispebjerg Hospital, Copenhagen, Denmark	September 1, 1991-	524	276 (53%)	248 (47%)	NA	Exclusion: SAH	Community-based study, prospective, single-center

Nr.	1. Author, year	2. Study center, country	3. Study period	3. N	4. Women, n (%)	5. Men, n (%)	6. Mean age women/men, y	7. Stroke subtype	8. Study design
			July 31, 1993						
47	Pollak, 2017	The National Acute Stroke Israeli (NASIS) Registry, 28 hospitals in Israel	April–May 2013	2,151	1,207 (56%)	944 (44%)	NA	Exclusion: SAH	Stroke registry, prospective, multicenter
48	Porteno y, 1984	Two teaching hospitals of the Albert Einstein College of Medicine, New York, US	NA	215	105 (49%)	110 (51%)	NA	Bland infarct, parenchymal hemorrhage, transient ischemic attacks, and lacunar infarcts	Cohort study, prospective, multicenter
49	Rathore, 2002	Atherosclerosis Risk in Communities, 4 communities in the US (Washington County, Maryland; northwest suburbs of Minneapolis, Minnesota; Forsyth County, North Carolina; and Jackson, Mississippi)	1986–1989	474	250 (53%)	224 (47%)	NA	TIA, cerebral hemorrhage, cerebral infarction (thrombotic or embolic), or SAH	Cohort study, retrospective, multicenter
50	Roquer, 2003	Hospital del Mar, Barcelona, Spain	Decembe r 1995-January 2002	1,581	809 (51%)	772 (49%)	75/69	All subtypes	Cohort study, prospective, single-center
51	Stuart-Shor, 2009	Beth Israel Deaconess Medical Center in Israel	April 1, 1999-Decembe r 31, 2004	1,107	499 (45%)	608 (55%)	NA	Ischemic stroke	Cohort study, prospective, single-center
52	Tentsch ert, 2005	Vienna Stroke Registry, 8	October 1998-	2,196	1,230 (56%)	966 (44%)	73/66 (median)	Ischemic stroke or TIA	Hospital-based stroke registry, prospective, multicenter

Nr.	1. Author, year	2. Study center, country	3. Study period	3. N	4. Women, n (%)	5. Men, n (%)	6. Mean age women/men, y	7. Stroke subtype	8. Study design
		neurological departments in Vienna, Austria	December 2001						
53	Tsouli, 2009	Athens Stroke Registry, Athens University Medical School, Athens, Greece	January 1995-December 2007	2,297	1,455 (63%)	842 (37%)	NA	TIA, SAH, and recurrent stroke excluded	Observational stroke databank, hospital-based study, prospective, single-center
54	van Os, 2016	Dutch Acute Stroke Study (DUST): 5 hospitals in The Netherlands & Leiden Stroke Cohort: one hospital, Leiden, The Netherlands	DUST (May 2012-August 2013) and Leiden Stroke Cohort (August 2013-March 2014)	284	109 (38%)	175 (62%)	NA	Ischemic stroke	DUST (cohort study, prospective, multicenter). Leiden Stroke Cohort (cohort study, prospective, single-center)
55	Vestergaard, 1993	Farsoe Hospital (a local hospital) and Aalborg Hospital (a regional hospital), Denmark	February 1, 1991-January 31, 1992	238	106 (45%)	132 (55%)	NA	ICH, infarction, and lacunar infarction	Cohort study, prospective, multicenter
56	Watila, 2011	University of Maiduguri Teaching Hospital, Maiduguri, Borno State, Nigeria	January2005 - December 2009	91	30 (37%)	61 (63%)	56/56	SAH, subdural hematoma, and history of stroke were excluded	Hospital based study, prospective, single-center
57	Wiszniewska, 2011	2nd Department of Neurology, Institute of Psychiatry and Neurology, Warsaw, Poland	1995-2007	2,534	1,155 (46%)	1,379 (54%)	74/69	Ischemic stroke	Stroke registry, prospective, single-center

Nr.	1. Author, year	2. Study center, country	3. Study period	3. N	4. Women, n (%)	5. Men, n (%)	6. Mean age women/men, y	7. Stroke subtype	8. Study design
58	Wolf, 1992	Framingham Study, US	Three successive decades beginning in 1953, 1963, and 1973	170	85 (50%)	85 (50%)	NA	Stroke and TIA	Cohort study, prospective, multicenter
59	Yeşilot, 2011	Istanbul Medical School Stroke Registry, Istanbul Medical School (a university neurology clinic), Istanbul, Turkey	March 1, 1994-March 31, 1999	1,522	751 (49%)	771 (51%)	65/62	Patients with SAH were excluded	Registry cohort, prospective, single-center
60	Yu, 2019	Substudy of SpecTRA (Spectrometry for Transient Ischemic Attack Rapid Assessment), two academic emergency departments, Canada	December 2013-March 2017	1,648	770 (47%)	878 (53%)	71/69 (median)	Acute transient or minor neurologic events	Cohort study, prospective, multicenter

Abbreviations
N=number of patients; RCT=Randomized Controlled Trial; US=United States; TIA=transient ischemic attack; ICH=intracerebral hemorrhage; SAH=subarachnoid hemorrhage; NA=not available.

Supplement Table II. Reported symptoms in included studies

Nr.	**Author, year**	**Symptoms reported**
1	Abadie, 2014	headache
2	Acciarresi, 2014	Dysarthria, disorders of visual functions, headache, consciousness disorders
3	Ahmadi Aghangar, 2015	Headache
4	Alves, 2012	Decreased LOC at presentation, headache
5	Arboix, 2001	Altered consciousness, homonymous hemianopia, ataxia
6	Aziz, 2016	Hemiparesis, headache, vertigo and nausea/vomiting*, visual alteration
7	Barrett, 2007	Weakness, visual deficits
8	Becker, 1986	Disoriented/lethargic, stuporous or comatose
9	Bersano, 2009	Aphasia
10	Brust, 1976	Aphasia
11	Chen, 2013	Headache
12	Di Carlo, 2003	Confusion, coma, weakness, aphasia, dysarthria
13	Elhfnawy, 2020	Vertigo
14	Engelter, 2006	Aphasia
15	Ferro, 1995	Headache
16	Ferro, 1997	Aphasia
17	Foerch, 2007	Reduced consciousness, any paresis
18	Gall, 2010	Facial weakness, dysarthria, headache, visual deficit, loss of consciousness, confusion, vertigo and nausea/vomiting, ataxia
19	Gargano, 2009	Weakness, facial droop, confusion, loss of consciousness, vision problems, imbalance, dizziness
20	Gialanella, 2011	Aphasia
21	Glader, 2003	Impairment of consciousness
22	Godefroy, 2002	Aphasia
23	Grindal, 1974	Headache
24	Hier, 1994	Aphasia
25	Inatomi, 2008	Aphasia
26	Jerath, 2011	**Signs: loss of consciousness, visual field loss, dysarthria, ataxia.<br>Symptoms: generalized weakness and shortness of breath or cyanotic taken together, headache, disorientation and confusion or difficulty with memory combined with mental status change, double vision, visual disturbances (combined with visual field loss as a sign), vertigo with nausea/vomiting
27	Kadojić, 2012	Aphasia
28	Kapral, 2005	Unconsciousness, headache, weakness, aphasia
29	Kes, 2016	Loss of consciousness, headache

Nr.	**Author, year**	**Symptoms reported**
30	Khan, 2018	Headache, coma, dysarthria†, aphasia‡
31	Kumral, 1995	Headache
32	Labiche, 2002	Gait, imbalance, dysarthria, aphasia, facial droop, change in level of consciousness, vision, dizziness-vertigo, unclassifiable neurologic symptoms (e.g., nausea, non-focal weakness, hiccups) combined with nonspecific symptoms (e.g., chest pain, shortness of breath, palpitations)
33	Lai, 2005	Blurred or decreased vision
34	Laska, 2001	Aphasia
35	Leira, 2002	Headache
36	Leira, 2005	Headache
37	Li, 2016	Any weakness, facial weakness, aphasia, dysarthria, monocular blindness and visual field defect combined, headache
38	Lisabeth, 2009	Hemi-body numbness, hemiparesis, aphasia, dysarthria, visual disturbance excluding diplopia, diplopia, facial weakness, vertigo, light-headedness, face or hemi-body pain, headache, other neurologic symptoms, non-neurologic symptoms, discoordination/ataxia
39	Maino, 2013	Headache
40	Medlin, 2020	Paresis, dysarthria, visual field defect, aphasia, decreased level of vigilance
41	Melo, 1996	Headache
42	Mitsias, 2006	Headache
43	Mochari-Greenberger, 2015	Other neurological symptoms, paresis/hemiparesis
44	Niewada, 2005	Drowsy, unconscious
45	Park, 2013	Altered mentality, gait disturbance, dizziness
46	Pedersen, 1996	Disorientation
47	Pollak, 2017	Headache
48	Portenoy, 1984	Headache
49	Rathore, 2002	Headache, vertigo, hemianopia, diplopia, paresis at any site, paresis of the face, gait disturbance
50	Roquer, 2003	Aphasic disorder, visual deficit
51	Stuart-Shor, 2009	Weakness, nausea, headache, change in vision
52	Tentschert, 2005	Headache
53	Tsouli, 2009	Aphasia
54	van Os, 2016	Headache
55	Vestergaard, 1993	Headache
56	Watila, 2011	Right and left hemiparesis combined, facial weakness, coma, headache, vomiting, hemianopsia, aphasia, dysarthria
57	Wiszniewska, 2011	Drowsy, stupor and coma combined
58	Wolf, 1992	Unconscious
59	Yeşilot, 2011	Stupor/coma, aphasia

Nr.	**Author, year**	**Symptoms reported**
60	Yu, 2019	Headache, vertigo (including spinning of vision itself)/vertigo (without visual spinning) combined with nausea/vomiting, dizziness, unsteady gait, confusion and disorientation combined, shortness of breath, chest pain, and palpitations with event combined

* Nausea or vomiting reported by Aziz et al. was not included in our meta-analysis since the number of patients reporting nausea or vomiting did not seem to be in line with the percentages.

† Both symptoms and signs reported. Whenever both symptoms and signs fitted within our a priori defined symptom categories, we chose to include data regarding signs collected from the neurological examination above symptoms collected from the medical records.

‡ Dysarthria and aphasia were only assessed in patients with ischemic stroke and not in patients with hemorrhagic stroke.

Supplement Table III. Risk of bias assessment of included studies

Nr.	Author, year	1. Diagnosis validated	2. Symptom assessment	3. Adjustment for confounders	4. Stroke specific inclusion criteria
1	Abadie, 2014	Low	High	High	Low
2	Acciarresi, 2014	Low	Low	High	Low
3	Ahmadi Aghangar, 2015	Low	High	High	High
4	Alves, 2012	Low	High	High	High
5	Arboix, 2001	Low	Low	High	Low
6	Aziz, 2016	Low	Low	High	High
7	Barrett, 2007	Low	Low	High	High
8	Becker, 1986	High	Low	High	Low
9	Bersano, 2009	Low	Low	High	Low
10	Brust, 1976	High	Low	High	Possible
11	Chen, 2013	Low	Low	High	High
12	Di Carlo, 2003	Low	High	High	Low
13	Elhfnawy, 2020	Low	Low	High	High
14	Engelter, 2006	Low	Low	High	High
15	Ferro, 1995	Low	Low	High	High
16	Ferro, 1997	Low	Low	High	High
17	Foerch, 2007	High	Low	High	Low
18	Gall, 2010	Low	Low	High	Low
19	Gargano, 2009	Low	High	High	Low
20	Gialanella, 2011	Low	Low	High	Low
21	Glader, 2003	Low	Possible	Low	Low
22	Godefroy, 2002	Possible	High	High	Low
23	Grindal, 1974	High	Possible	High	High
24	Hier, 1994	Low	High	High	Low
25	Inatomi, 2008	Low	Low	High	High
26	Jerath, 2011	Low	Low	High	High
27	Kadojić, 2012	Low	Low	High	Low
28	Kapral, 2005	Low	Low	High	Low
29	Kes, 2016	Low	High	High	Low
30	Khan, 2018	Low	Possible	High	High
31	Kumral, 1995	Low	High	High	Low
32	Labiche, 2002	Low	High	Low	Low

Nr.	**Author, year**	**1. Diagnosis validated**	**2. Symptom assessment**	**3. Adjustment for confounders**	**4. Stroke specific inclusion criteria**
33	Lai, 2005	Low	Low	High	Low
34	Laska, 2001	Low	Low	High	Low
35	Leira, 2002	Low	Possible	High	High
36	Leira, 2005	Low	Low	High	High
37	Li, 2016	High	Low	High	High
38	Lisabeth, 2009	Low	High	High	High
39	Maino, 2013	Possible	Low	High	High
40	Medlin, 2020	Low	Possible	High	High
41	Melo, 1996	Low	Low	High	High
42	Mitsias, 2006	Low	Low	High	High
43	Mochari-Greenberger, 2015	High	Low	Low	Low
44	Niewada, 2005	Low	Low	High	High
45	Park, 2013	Low	Low	High	High
46	Pedersen, 1996	Low	Low	High	Low
47	Pollak, 2017	Low	Low	High	Low
48	Portenoy, 1984	Low	Low	High	Low
49	Rathore, 2002	Low	Low	High	Low
50	Roquer, 2003	Low	Low	High	Low
51	Stuart-Shor, 2009	High	Low	Low	High
52	Tentschert, 2005	Low	Low	High	High
53	Tsouli, 2009	Low	Low	High	Low
54	van Os, 2016	Low	Low	High	High
55	Vestergaard, 1993	Low	Low	High	Low
56	Watila, 2011	Low	Possible	High	Low
57	Wiszniewska, 2011	Low	Low	High	High
58	Wolf, 1992	Low	Low	High	Low
59	Yeşilot, 2011	Low	Low	High	Low
60	Yu, 2019	Low	Low	High	High

Supplement Figure I. Forest plot of non-focal symptoms

Author(s) and Year	Women Events	Women Total	Men Events	Men Total	OR [95% CI]
Headache including migraine					
Khan, 2018	49	73	77	197	3.18 [1.81, 5.60]
Watila, 2011	6	30	10	61	1.28 [0.42, 3.92]
van Os , 2016	49	109	60	175	1.57 [0.96, 2.55]
Ahmadi Aghangar, 2015	18	124	22	139	0.90 [0.46, 1.78]
Abadie, 2014	129	753	124	634	0.85 [0.65, 1.12]
Maino, 2013	162	865	258	1608	1.21 [0.97, 1.50]
Chen , 2013	372	4503	476	7020	1.24 [1.08, 1.43]
Leira, 2002	12	76	61	165	0.32 [0.16, 0.64]
Vestergaard, 1993	39	106	26	132	2.37 [1.32, 4.25]
Mitsias, 2006	53	164	65	211	1.07 [0.69, 1.66]
Melo, 1996	81	119	84	170	2.18 [1.34, 3.56]
Leira, 2005	33	81	32	108	1.63 [0.89, 2.99]
Grindal, 1974	26	107	14	53	0.89 [0.42, 1.90]
Ferro, 1995	25	82	35	123	1.10 [0.60, 2.03]
Kumral, 1995	204	944	252	1562	1.43 [1.17, 1.76]
Stuart−Shor, 2009	64	608	56	499	0.93 [0.64, 1.36]
Kes, 2016	48	210	38	186	1.15 [0.71, 1.87]
Tentschert, 2005	288	979	300	1217	1.27 [1.05, 1.54]
Portenoy, 1984	47	110	24	105	2.52 [1.39, 4.55]
Alves, 2012	79	173	83	191	1.09 [0.72, 1.65]
Pollak, 2016	102	944	100	1207	1.34 [1.00, 1.79]
Yu, 2019	285	770	265	878	1.36 [1.11, 1.67]
Rathore, 2002	71	224	60	250	1.47 [0.98, 2.20]
Kapral, 2005	474	1527	396	1796	1.59 [1.36, 1.86]
Lisabeth, 2009	31	224	28	237	1.20 [0.69, 2.07]
Gall, 2010	141	603	127	495	0.88 [0.67, 1.17]
Jerath, 2011	43	268	31	181	0.92 [0.56, 1.53]
Acciarresi, 2014	64	811	93	1072	0.90 [0.65, 1.26]
Li, 2016	612	2912	468	3079	1.48 [1.30, 1.70]
Aziz, 2016	494	2138	606	2624	1.00 [0.87, 1.15]
RE Model for Subgroup (Q = 95.17, df = 29, p = 0.00; I^2 = 75.2%)					1.24 [1.11, 1.39]
Change in level of consciousness/mental status c hange (GCS<=14)					
Medlin, 2020	214	1712	241	2191	1.16 [0.95, 1.41]
Jerath, 2011	238	268	107	181	5.49 [3.39, 8.88]
Pedersen, 1996	63	276	36	248	1.74 [1.11, 2.74]
Glader, 2003	2096	9477	1568	9719	1.48 [1.37, 1.59]
Wiszniewska , 2011	236	1374	146	1151	1.43 [1.14, 1.78]
Niewada, 2005	2001	8003	1761	9367	1.44 [1.34, 1.55]
Becker , 1986	560	1779	451	1555	1.12 [0.97, 1.30]
Arboix, 2001	219	967	186	1033	1.33 [1.07, 1.66]
Labiche, 2002	114	657	56	467	1.54 [1.09, 2.17]
Alves, 2012	95	173	108	191	0.94 [0.62, 1.42]
Foerch, 2007	6475	26319	4227	27095	1.77 [1.69, 1.84]
Gargano, 2009	99	1041	73	881	1.16 [0.85, 1.60]
Di Carlo , 2003	683	2260	652	2239	1.05 [0.93, 1.20]
Gall , 2010	99	461	71	389	1.22 [0.87, 1.72]
Park, 2013	427	2830	437	3805	1.37 [1.19, 1.58]
Yu, 2019	148	770	192	878	0.85 [0.67, 1.08]
Acciarresi, 2014	208	811	188	1072	1.62 [1.30, 2.03]
RE Model for Subgroup (Q = 167.33, df = 16, p = 0.00; I^2 = 95.0%)					1.38 [1.19, 1.61]
Change in level of consciousness/mental status c hange (GCS<=8)					
Watila, 2011	9	30	3	61	8.29 [2.05, 33.56]
Khan, 2018	11	73	33	197	0.88 [0.42, 1.85]
Becker, 1986	404	1779	294	1555	1.26 [1.06, 1.49]
Yesilot, 2011	69	751	53	771	1.37 [0.94, 1.99]
Wiszniewska, 2011	185	1374	88	1151	1.88 [1.44, 2.45]
Di Carlo , 2003	229	2260	157	2239	1.50 [1.21, 1.85]
Niewada , 2005	136	8003	113	9367	1.42 [1.10, 1.82]
Kapral, 2005	107	1527	108	1796	1.18 [0.89, 1.55]
Wolf, 1992	12	85	15	85	0.77 [0.34, 1.75]
Gargano, 2009	74	1041	54	881	1.17 [0.82, 1.68]
Gall , 2010	202	535	120	459	1.71 [1.31, 2.25]
Jerath, 2011	88	268	50	181	1.28 [0.85, 1.94]
Kes, 2016	25	210	20	186	1.12 [0.60, 2.09]
RE Model for Subgroup (Q = 21.48, df = 12, p = 0.04; I^2 = 27.0%)					1.39 [1.25, 1.55]
Light−headedness					
Yu, 2019	74	770	75	878	1.14 [0.81, 1.59]
Lisabeth, 2009	29	224	36	237	0.83 [0.49, 1.41]
RE Model for Subgroup (Q = 0.98, df = 1, p = 0.32; I^2 = 0.0%)					1.04 [0.78, 1.38]
Atypical symptoms					
Yu, 2019	88	770	76	878	1.36 [0.99, 1.88]
Labiche, 2002	283	657	181	467	1.20 [0.94, 1.52]
Stuart−Shor, 2009	521	608	427	499	1.01 [0.72, 1.42]
Gargano, 2009	347	1041	304	881	0.95 [0.79, 1.15]
Di Carlo, 2003	981	2260	1037	2239	0.89 [0.79, 1.00]
Kapral, 2005	1023	1527	1258	1796	0.87 [0.75, 1.01]
Barrett, 2007	158	229	164	276	1.52 [1.05, 2.20]
Jerath, 2011	98	268	47	181	1.64 [1.09, 2.49]
Li, 2016	1660	2912	1823	3079	0.91 [0.82, 1.01]
RE Model for Subgroup (Q = 24.48, df = 8, p = 0.00; I^2 = 77.2%)					1.06 [0.92, 1.22]
Aspecific neurological or other neur ological symptoms					
Yu, 2019	189	770	205	878	1.07 [0.85, 1.34]
Lisabeth, 2009	44	224	40	237	1.20 [0.75, 1.93]
Mochari−Greenberger, 2015	64326	201017	65268	197781	0.96 [0.94, 0.97]
Gargano, 2009	72	1041	79	881	0.75 [0.54, 1.05]
Park, 2013	387	2830	561	3805	0.92 [0.80, 1.05]
RE Model for Subgroup (Q = 4.13, df = 4, p = 0.39; I^2 = 0.1%)					0.96 [0.94, 0.97]
RE Model for All Studies (Q = 1263.27, df = 75, p = 0.00; I^2 = 91.9%)					1.24 [1.16, 1.33]

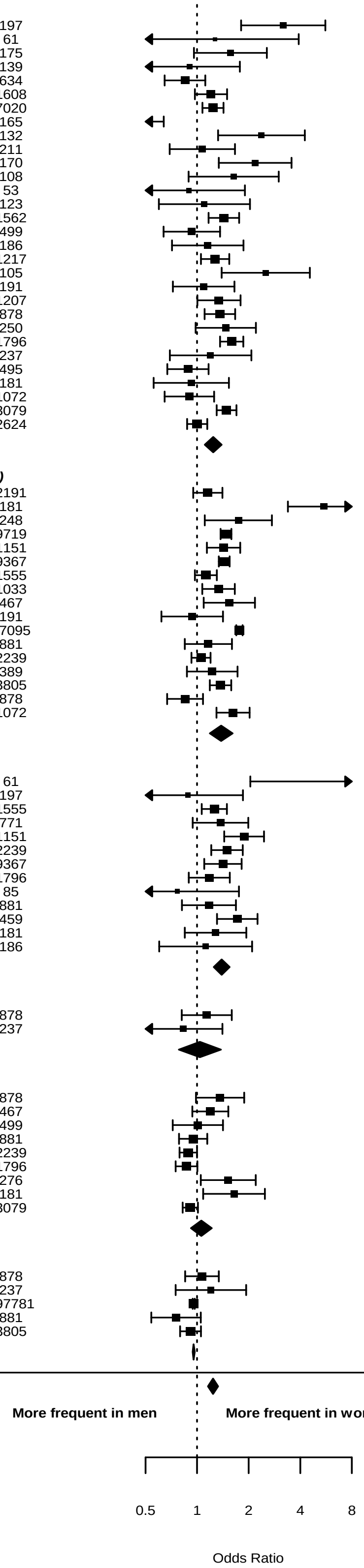

Supplement Figure II. Sensitivity analysis of nonspecific neurological or other neurological symptoms only (Mochari-Greenberger et al. excluded)

	Women		Men		
**Author(s) and Year**	**Events**	**Total**	**Events**	**Total**	**OR [95% CI]**
Park, 2013	387	2830	561	3805	0.92 [0.80, 1.05]
Gargano, 2009	72	1041	79	881	0.75 [0.54, 1.05]
Lisabeth, 2009	44	224	40	237	1.20 [0.75, 1.93]
Yu, 2019	189	770	205	878	1.07 [0.85, 1.34]
RE Model for Subgroup (Q = 4.07, df = 3, p = 0.25; I^2 = 8.9%)					0.95 [0.84, 1.07]

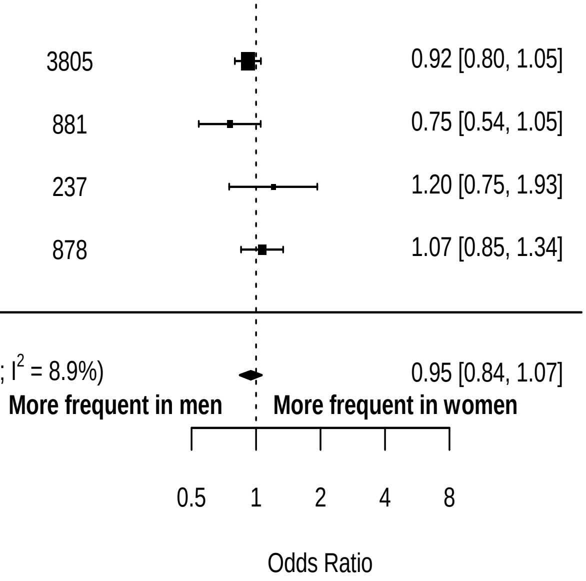

Supplement Figure III. Forest plot of focal symptoms

Author(s) and Year	Women Events	Women Total	Men Events	Men Total	OR [95% CI]
Gait disturbance					
Stuart−Shor, 2009	182	608	169	499	0.83 [0.65, 1.08]
Rathore , 2002	15	224	36	250	0.43 [0.23, 0.80]
Yu , 2019	227	770	288	878	0.86 [0.69, 1.06]
Labiche, 2002	50	657	50	467	0.69 [0.46, 1.04]
Park, 2012	322	2830	431	3805	1.01 [0.86, 1.17]
RE Model for Subgroup (Q = 9.52, df = 4, p = 0.05; I^2 = 53.5%)					1.05 [0.92, 1.19]
Imbalance					
Labiche, 2002	100	657	93	467	0.72 [0.53, 0.99]
Gargano, 2009	23	1041	32	881	0.60 [0.35, 1.03]
RE Model for Subgroup (Q = 0.34, df = 1, p = 0.56; I^2 = 0.0%)					0.85 [0.23, 3.14]
Vertigo with or without nausea/v omiting					
Elhfnawy, 2020	15	21	17	38	3.09 [0.99, 9.68]
Yu, 2019	302	770	304	878	1.22 [1.00, 1.49]
Labiche, 2002	53	657	45	467	0.82 [0.54, 1.25]
Rathore, 2002	4	224	6	250	0.74 [0.21, 2.65]
Lisabeth, 2009	18	224	12	237	1.64 [0.77, 3.48]
Gall, 2010	42	417	39	365	0.94 [0.59, 1.48]
Jerath, 2011	43	268	35	181	0.80 [0.49, 1.30]
Aziz, 2016	351	2138	489	2624	0.86 [0.74, 1.00]
RE Model for Subgroup (Q = 14.73, df = 7, p = 0.04; I^2 = 44.0%)					1.23 [1.13, 1.34]
Facial weakness					
Watila, 2011	22	30	31	61	2.66 [1.03, 6.90]
Rathore, 2002	122	224	137	250	0.99 [0.69, 1.42]
Gargano, 2009	86	1041	54	881	1.38 [0.97, 1.96]
Labiche, 2002	60	657	40	467	1.07 [0.71, 1.63]
Lisabeth, 2009	30	224	35	237	0.89 [0.53, 1.51]
Li, 2016	915	2912	866	3079	1.17 [1.05, 1.31]
Gall, 2010	269	535	234	459	0.97 [0.76, 1.25]
RE Model for Subgroup (Q = 7.47, df = 6, p = 0.28; I^2 = 0.0%)					0.90 [0.81, 1.00]
Paresis/hemiparesis					
Watila, 2011	27	30	56	61	0.80 [0.18, 3.61]
Medlin, 2020	1408	1757	1709	2236	1.24 [1.07, 1.45]
Jerath, 2011	89	268	38	181	1.87 [1.21, 2.90]
Rathore, 2002	185	224	202	250	1.13 [0.71, 1.80]
Foerch, 2007	19661	26319	19726	27095	1.10 [1.06, 1.15]
Lisabeth, 2009	86	224	93	237	0.96 [0.66, 1.40]
Aziz, 2016	1592	2138	1992	2624	0.93 [0.81, 1.06]
RE Model for Subgroup (Q = 15.39, df = 6, p = 0.02; I^2 = 72.6%)					0.73 [0.54, 0.97]
Discoordination/ataxia					
Arboix, 2001	40	967	70	1033	0.59 [0.40, 0.88]
Jerath, 2011	124	268	94	181	0.80 [0.55, 1.16]
Lisabeth, 2009	103	224	106	237	1.05 [0.73, 1.52]
Gall, 2010	58	535	83	459	0.55 [0.38, 0.79]
RE Model for Subgroup (Q = 7.38, df = 3, p = 0.06; I^2 = 59.1%)					1.12 [0.96, 1.30]
Dysarthria					
Watila, 2011	4	30	4	61	2.19 [0.51, 9.45]
Khan, 2018	6	60	39	157	0.34 [0.13, 0.84]
Medlin, 2020	889	1757	1126	2236	1.01 [0.89, 1.14]
Godefroy, 2002	9	143	13	165	0.79 [0.33, 1.90]
Labiche, 2002	100	657	73	467	0.97 [0.70, 1.35]
Di Carlo, 2003	705	2260	811	2239	0.80 [0.71, 0.90]
Lisabeth, 2009	52	224	70	237	0.72 [0.48, 1.09]
Gall, 2010	192	446	197	388	0.73 [0.56, 0.96]
Jerath, 2011	95	268	69	181	0.89 [0.60, 1.32]
Acciarresi, 2014	302	811	402	1072	0.99 [0.82, 1.19]
Li, 2016	976	2912	1016	3079	1.02 [0.92, 1.14]
RE Model for Subgroup (Q = 21.59, df = 10, p = 0.02; I^2 = 48.6%)					1.14 [1.04, 1.24]
Aphasia					
Engelter , 2006	53	155	27	114	1.67 [0.97, 2.89]
Watila, 2011	6	30	32	61	0.23 [0.08, 0.63]
Khan, 2018	21	60	57	157	0.94 [0.51, 1.76]
Medlin, 2020	643	1757	698	2236	1.27 [1.11, 1.45]
Inatomi, 2008	62	324	68	531	1.61 [1.11, 2.35]
Tsouli, 2009	348	842	458	1455	1.53 [1.29, 1.83]
Bersano, 2009	1313	4192	1161	4656	1.37 [1.25, 1.51]
Godefroy, 2002	98	143	109	165	1.12 [0.69, 1.80]
Laska, 2001	19	57	17	49	0.94 [0.42, 2.11]
Brust, 1976	87	472	90	378	0.72 [0.52, 1.01]
Kadojic, 2012	40	83	35	94	1.57 [0.86, 2.86]
Ferro, 1997	68	128	147	295	1.14 [0.75, 1.73]
Gialanella, 2010	71	127	60	135	1.58 [0.97, 2.58]
Hier, 1994	218	963	163	842	1.22 [0.97, 1.53]
Labiche, 2002	73	657	49	467	1.07 [0.73, 1.56]
Di Carlo, 2003	786	2260	679	2239	1.23 [1.08, 1.39]
Roquer, 2003	223	772	175	809	1.47 [1.17, 1.85]
Kapral, 2005	397	1527	467	1796	1.00 [0.86, 1.17]
Lisabeth, 2009	58	224	62	237	0.99 [0.65, 1.50]
Yesilot, 2011	215	751	167	771	1.45 [1.15, 1.83]
Jerath, 2011	126	268	67	181	1.51 [1.03, 2.22]
Li , 2016	676	2912	647	3079	1.14 [1.01, 1.28]
RE Model for Subgroup (Q = 52.93, df = 21, p = 0.00; I^2 = 59.6%)					1.00 [0.83, 1.20]
Diplopia					
Rathore, 2002	12	224	14	250	0.95 [0.43, 2.11]
Lisabeth, 2009	12	224	5	237	2.63 [0.91, 7.58]
Jerath, 2011	5	268	13	181	0.25 [0.09, 0.70]
RE Model for Subgroup (Q = 9.80, df = 2, p = 0.01; I^2 = 81.8%)					0.69 [0.53, 0.90]
Other focal visual disturbances					
Watila, 2011	2	30	4	61	1.02 [0.18, 5.90]
Medlin, 2020	666	1757	739	2236	1.24 [1.09, 1.41]
Stuart−Shor, 2009	160	608	121	499	1.12 [0.85, 1.47]
Gargano, 2009	37	1041	42	881	0.74 [0.47, 1.16]
Arboix, 2001	200	967	171	1033	1.31 [1.05, 1.65]
Labiche, 2002	41	657	23	467	1.28 [0.76, 2.17]
Rathore, 2002	29	224	40	250	0.78 [0.47, 1.31]
Roquer, 2003	133	772	111	809	1.31 [1.00, 1.72]
Lai, 2005	38	245	29	214	1.17 [0.69, 1.97]
Barrett, 2007	46	229	53	276	1.06 [0.68, 1.64]
Lisabeth, 2009	35	224	44	237	0.81 [0.50, 1.32]
Gall, 2010	154	535	104	459	1.38 [1.03, 1.84]
Jerath, 2011	79	268	61	181	0.82 [0.55, 1.23]
Acciarresi, 2014	172	811	192	1072	1.23 [0.98, 1.55]
Li , 2016	271	2912	348	3079	0.81 [0.69, 0.96]
Aziz, 2016	112	2138	181	2624	0.75 [0.59, 0.95]
RE Model for Subgroup (Q = 41.09, df = 15, p = 0.00; I^2 = 62.8%)					0.83 [0.70, 0.99]
RE Model for All Studies (Q = 294.19, df = 84, p = 0.00; I^2 = 78.8%)					1.03 [0.97, 1.09]

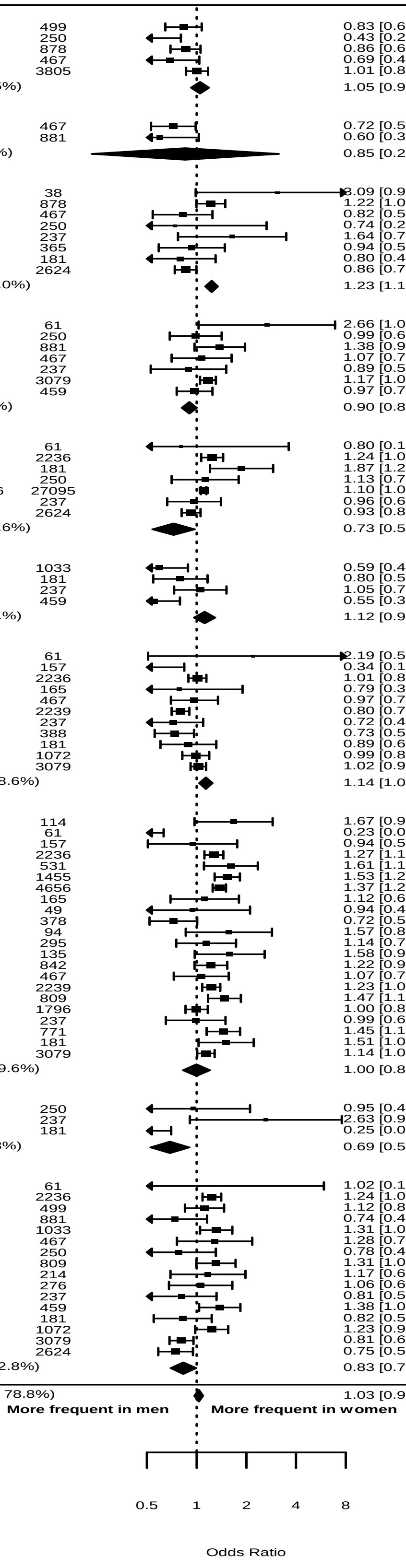

Supplement Figure IV. Forest plot of the subgroup analysis for non-focal symptoms in patients with ischemic stroke

Author(s) and Year	Women Events	Women Total	Men Events	Men Total	OR [95% CI]
Headache including migraine					
Khan, 2018	41	60	49	157	4.76 [2.51, 9.02]
Abadie, 2014	82	639	78	546	0.88 [0.63, 1.23]
Mitsias, 2006	53	164	65	211	1.07 [0.69, 1.66]
van Os , 2016	49	109	60	175	1.57 [0.96, 2.55]
Ahmadi Aghangar, 2015	18	124	22	139	0.90 [0.46, 1.78]
Chen , 2013	372	4503	476	7020	1.24 [1.08, 1.43]
Leira, 2002	12	76	61	165	0.32 [0.16, 0.64]
Ferro, 1995	25	82	35	123	1.10 [0.60, 2.03]
Stuart−Shor, 2009	64	608	56	499	0.93 [0.64, 1.36]
Jerath, 2011	43	268	31	181	0.92 [0.56, 1.53]
Aziz, 2016	494	2138	606	2624	1.00 [0.87, 1.15]
RE Model for Subgroup (Q = 42.04, df = 10, p = 0.00; I^2 = 90.1%)					1.08 [0.79, 1.49]
Change in level of consciousness/mental status change (GCS <=14)					
Medlin, 2020	214	1712	241	2191	1.16 [0.95, 1.41]
Wiszniewska , 2011	236	1374	146	1151	1.43 [1.14, 1.78]
Niewada, 2005	2001	8003	1761	9367	1.44 [1.34, 1.55]
Park, 2013	427	2830	437	3805	1.37 [1.19, 1.58]
RE Model for Subgroup (Q = 4.37, df = 3, p = 0.22; I^2 = 29.3%)					1.38 [1.27, 1.50]
Change in level of consciousness/mental status change (GCS <=8)					
Khan, 2018	5	60	15	157	0.86 [0.30, 2.48]
Wiszniewska, 2011	185	1374	88	1151	1.88 [1.44, 2.45]
Niewada , 2005	136	8003	113	9367	1.42 [1.10, 1.82]
Jerath, 2011	88	268	50	181	1.28 [0.85, 1.94]
RE Model for Subgroup (Q = 4.49, df = 3, p = 0.21; I^2 = 34.1%)					1.50 [1.20, 1.88]
Atypical symptoms					
Stuart−Shor, 2009	521	608	427	499	1.01 [0.72, 1.42]
Barrett, 2007	158	229	164	276	1.52 [1.05, 2.20]
Jerath, 2011	200	268	143	181	0.78 [0.50, 1.23]
RE Model for Subgroup (Q = 5.40, df = 2, p = 0.07; I^2 = 63.7%)					1.08 [0.75, 1.56]
RE Model for All Studies (Q = 85.15, df = 21, p = 0.00; I^2 = 85.0%)					1.20 [1.04, 1.38]

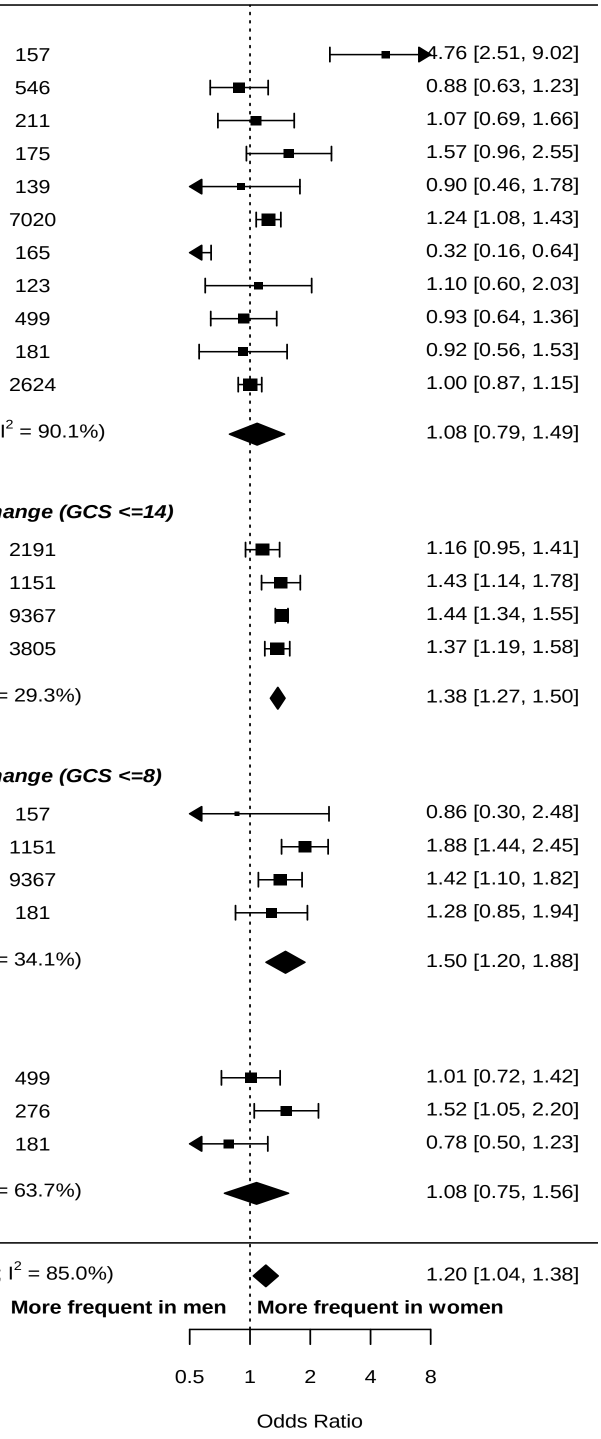

Supplement Figure V. Forest plot of the subgroup analysis for focal symptoms in patients with ischemic stroke

Author(s) and Year	Women Events	Women Total	Men Events	Men Total	OR [95% CI]
Dysarthria					
Jerath, 2011	95	268	69	181	0.89 [0.60, 1.32]
Khan, 2018	6	60	39	157	0.34 [0.13, 0.84]
Medlin, 2020	889	1757	1126	2236	1.01 [0.89, 1.14]
RE Model for Subgroup (Q = 5.66, df = 2, p = 0.06; I^2 = 74.8%)					0.81 [0.51, 1.27]
Gait disturbance					
Stuart−Shor, 2009	182	608	169	499	0.83 [0.65, 1.08]
Park, 2012	322	2830	431	3805	1.01 [0.86, 1.17]
RE Model for Subgroup (Q = 1.51, df = 1, p = 0.22; I^2 = 34.0%)					0.94 [0.79, 1.12]
Vertigo with or without nausea/vomiting					
Jerath, 2011	43	268	35	181	0.80 [0.49, 1.30]
Aziz, 2016	351	2138	489	2624	0.86 [0.74, 1.00]
Elhfnawy, 2020	15	21	17	38	3.09 [0.99, 9.68]
RE Model for Subgroup (Q = 4.88, df = 2, p = 0.09; I^2 = 0.0%)					0.87 [0.75, 1.00]
Paresis/hemiparesis					
Jerath, 2011	89	268	38	181	1.87 [1.21, 2.90]
Aziz, 2016	1592	2138	1992	2624	0.93 [0.81, 1.06]
Medlin, 2020	1408	1757	1709	2236	1.24 [1.07, 1.45]
RE Model for Subgroup (Q = 14.60, df = 2, p = 0.00; I^2 = 90.5%)					1.23 [0.86, 1.78]
Aphasia					
Engelter , 2006	53	155	27	114	1.67 [0.97, 2.89]
Inatomi, 2008	62	324	68	531	1.61 [1.11, 2.35]
Kadojic, 2012	40	83	35	94	1.57 [0.86, 2.86]
Ferro, 1997	68	128	147	295	1.14 [0.75, 1.73]
Jerath, 2011	126	268	67	181	1.51 [1.03, 2.22]
Hier, 1994	180	842	127	963	1.79 [1.40, 2.30]
Khan, 2018	21	60	57	157	0.94 [0.51, 1.76]
Medlin, 2020	643	1757	698	2236	1.27 [1.11, 1.45]
RE Model for Subgroup (Q = 9.41, df = 7, p = 0.22; I^2 = 31.3%)					1.43 [1.23, 1.65]
Other focal visual disturbance					
Stuart−Shor, 2009	160	608	121	499	1.12 [0.85, 1.47]
Barrett, 2007	46	229	53	276	1.06 [0.68, 1.64]
Jerath, 2011	79	268	61	181	0.82 [0.55, 1.23]
Aziz, 2016	112	2138	181	2624	0.75 [0.59, 0.95]
Medlin, 2020	666	1757	739	2236	1.24 [1.09, 1.41]
RE Model for Subgroup (Q = 14.84, df = 4, p = 0.01; I^2 = 68.8%)					1.00 [0.80, 1.23]
RE Model for All Studies (Q = 89.37, df = 23, p = 0.00; I^2 = 79.0%)					1.11 [0.99, 1.24]

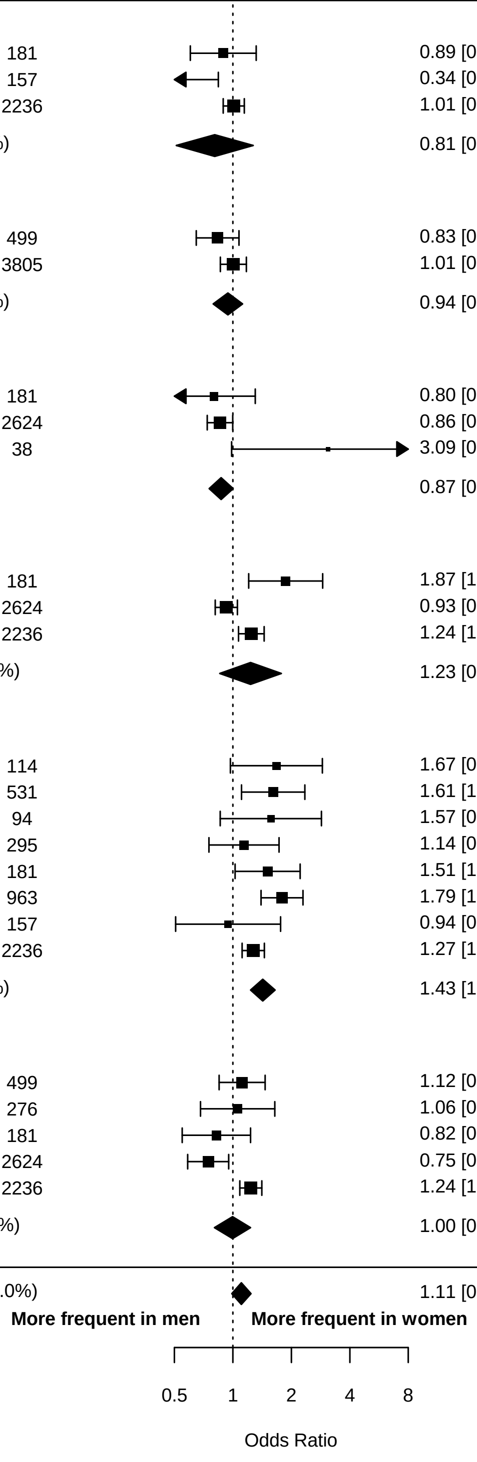

Supplement Figure VI. Forest plot of the subgroup analysis for headache in patients with a TIA

Author(s) and Year	Women Events	Women Total	Men Events	Men Total	OR [95% CI]
Li, 2015	612	2912	468	3079	1.48 [1.30, 1.70]
Yu, 2019	285	770	265	878	1.36 [1.11, 1.67]
Ferro, 1995	25	82	35	123	1.10 [0.60, 2.03]
Grindal, 1974	26	107	14	53	0.89 [0.42, 1.90]
RE Model for Subgroup (Q = 2.71, df = 3, p = 0.44; I^2 = 0.0%)					1.42 [1.27, 1.58]

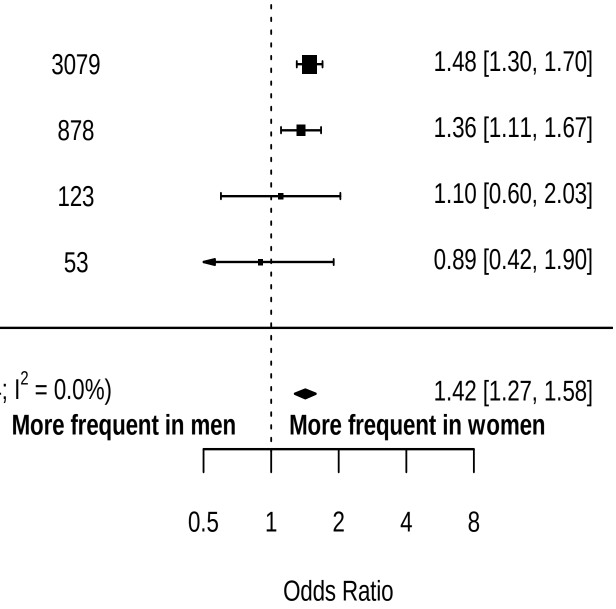

Supplement Figure VII. Forest plot of the subgroup analysis for headache in patients with ICH or SAH

Author(s) and Year	Women Events	Women Total	Men Events	Men Total	OR [95% CI]
Leira, 2005	33	81	32	108	1.63 [0.89, 2.99]
Melo, 1996	81	119	84	170	2.18 [1.34, 3.56]
Alves, 2012	79	173	83	191	1.09 [0.72, 1.65]
Abadie, 2014	47	114	46	87	0.63 [0.36, 1.10]
Khan, 2018	28	40	8	13	1.46 [0.40, 5.38]
RE Model for Subgroup (Q = 12.02, df = 4, p = 0.02; I^2 = 67.6%)					1.27 [0.80, 2.02]

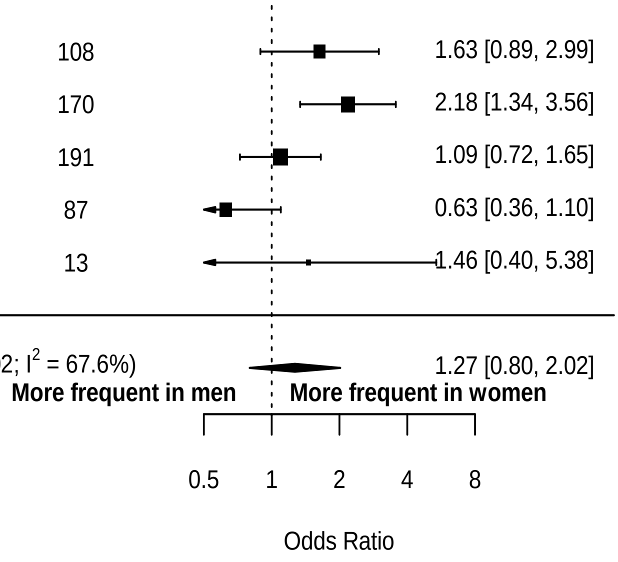

Supplement Figure VIII. Publication bias funnel plots

Funnel plots were produced for aphasia, other focal visual disturbances, minor change in level of consciousness/mental status change (GCS ≤14), coma or stupor (GCS ≤8), headache including migraine, and dysarthria. In general, inspection of the funnel plots showed a symmetric distribution, indicating a low chance of publication bias. Asymmetry was observed in the funnel plot for studies reporting on aphasia (Figure VIIIA), minor change in level of consciousness/mental status change (Figure VIIIC), and coma or stupor (Figure VIIID).

A. Funnel plot of studies for pooled OR of aphasia

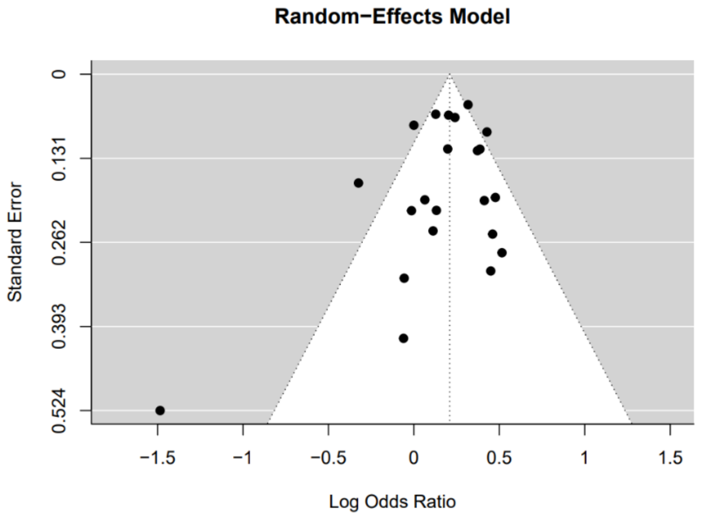

B. Funnel plot of studies for pooled OR of other focal visual disturbances

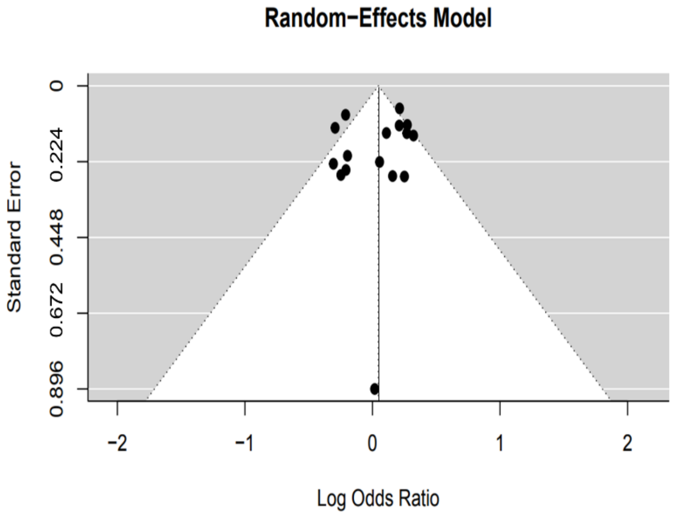

C. Funnel plot for pooled OR of minor change in level of consciousness/mental status change (GCS ≤14)

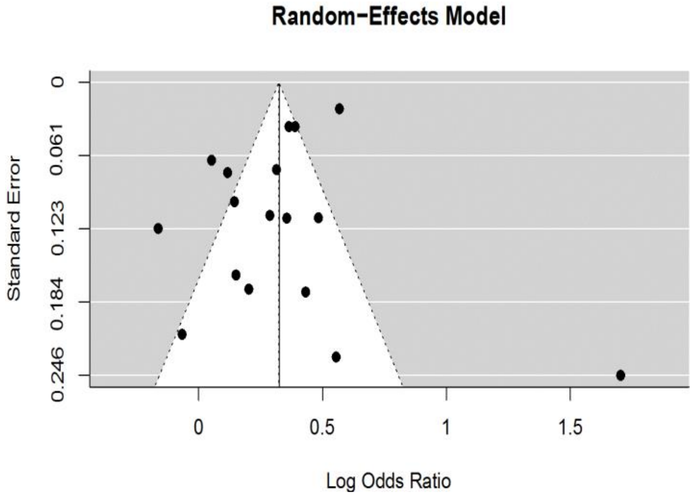

D. Funnel plot of studies for pooled OR of coma or stupor (GCS ≤8)

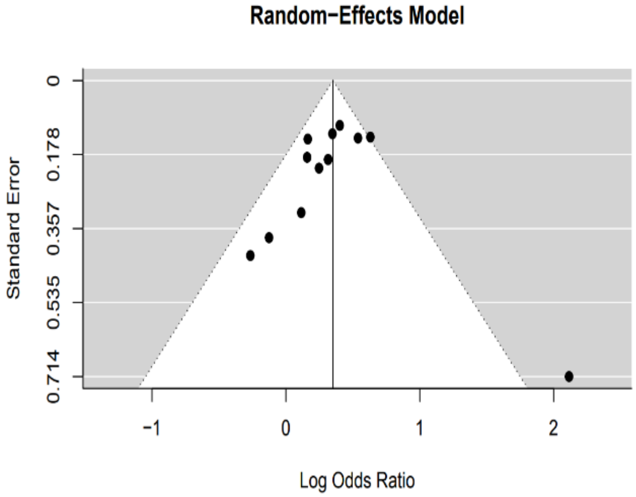

Supplement Figure VIII. Publication bias funnel plots (continued)

E. Funnel plot for pooled OR of headache including migraine

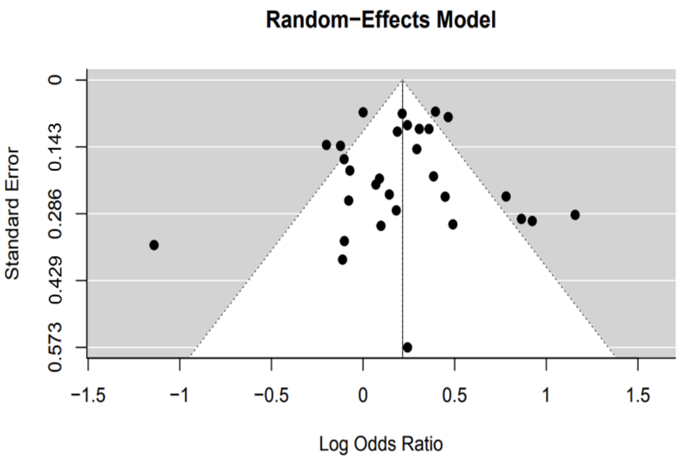

F. Funnel plot for pooled OR of dysarthria

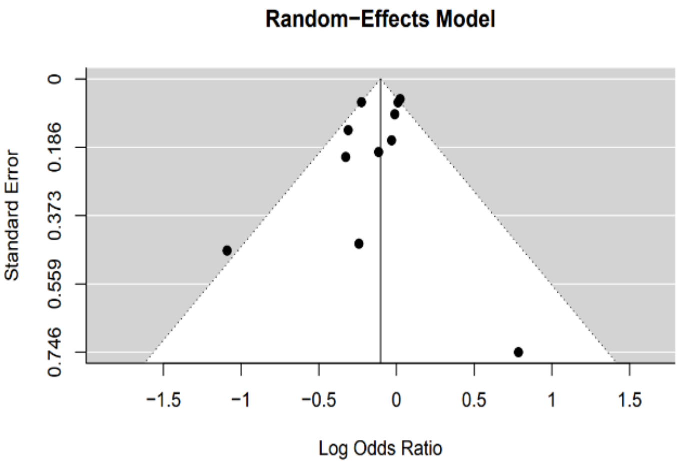

Supplement: Supplementary file 1 [file str-53-345-s001.pdf]
