# Supplementary figures and images for: Sex Differences in Presentation of Stroke: A Systematic Review and Meta-Analysis
Source: Stroke. 2021 Dec 14;53(2):345–54. doi: 10.1161/STROKEAHA.120.034040 (PMC8785516; doi:10.1161/STROKEAHA.120.034040)

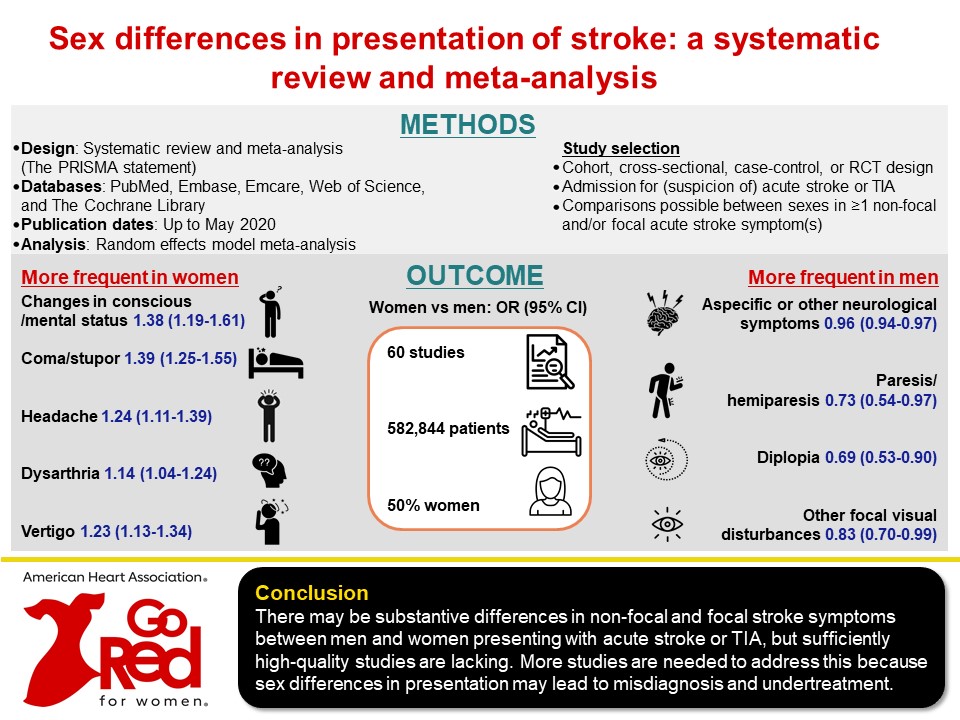

Supplement: Supplementary file 3 [file str-53-345-s003.jpg]
